# Electron–phonon coupling-assisted universal red luminescence of o-phenylenediamine-based carbon dots

**DOI:** 10.1038/s41377-022-00865-x

**Published:** 2022-06-06

**Authors:** Boyang Wang, Zhihong Wei, Laizhi Sui, Jingkun Yu, Baowei Zhang, Xiaoyong Wang, Shengnan Feng, Haoqiang Song, Xue Yong, Yuxi Tian, Bai Yang, Siyu Lu

**Affiliations:** 1 Green Catalysis Center, and College of Chemistry, Zhengzhou University, 450000 Zhengzhou, China; 2 Key Laboratory of Mesoscopic Chemistry of MOE, School of Chemistry and Chemical Engineering, Jiangsu Key Laboratory of Vehicle Emissions Control, Nanjing University, 210023 Nanjing, China; 3 State Key Lab of Molecular Reaction Dynamics, Dalian Institute of Chemical Physics, Chinese Academy of Sciences, 116023 Dalian, China; 4 Nanochemistry Department, Istituto Italiano di Tecnologia (IIT), via Morego 30, 16163 Genova, Italy; 5 School of Physics, National Laboratory of Solid State Microstructures, Collaborative Innovation Center of Advanced Microstructures, Nanjing University, 210093 Nanjing, China; 6 Department of Chemistry, University of Sheffield, Sheffield, S3 7HF UK; 7 State Key Lab of Supramolecular Structure and Materials, College of Chemistry, Jilin University, 130012 Changchun, China

**Keywords:** Quantum dots, Nanoparticles

## Abstract

Due to the complex core–shell structure and variety of surface functional groups, the photoluminescence (PL) mechanism of carbon dots (CDs) remain unclear. o-Phenylenediamine (oPD), as one of the most common precursors for preparing red emissive CDs, has been extensively studied. Interestingly, most of the red emission CDs based on oPD have similar PL emission characteristics. Herein, we prepared six different oPD-based CDs and found that they had almost the same PL emission and absorption spectra after purification. Structural and spectral characterization indicated that they had similar carbon core structures but different surface polymer shells. Furthermore, single-molecule PL spectroscopy confirmed that the multi-modal emission of those CDs originated from the transitions of different vibrational energy levels of the same PL center in the carbon core. In addition, the phenomenon of “spectral splitting” of single-particle CDs was observed at low temperature, which confirmed these oPD-based CDs were unique materials with properties of both organic molecules and quantum dots. Finally, theoretical calculations revealed their potential polymerization mode and carbon core structure. Moreover, we proposed the PL mechanism of red-emitting CDs based on oPD precursors; that is, the carbon core regulates the PL emission, and the polymer shell regulates the PL intensity. Our work resolves the controversy on the PL mechanism of oPD-based red CDs. These findings provide a general guide for the mechanism exploration and structural analysis of other types of CDs.

## Introduction

Carbon dots (CDs) are new carbon-based photoluminescence (PL) nanomaterials with a core–shell motif. Due to their fascinating advantage in chemical inertness, high quantum yields (QYs), high water solubility, thermal stability, and excellent biocompatibility^1^, CDs have aroused extensive attention in various research applications, for example, cancer diagnosis, phototherapy, and optoelectronic devices^2,3^. However, the underlying PL phenomena of CDs remain a mystery due to the polydispersity of the products and the difficulty in ascertaining their atomic structures.

At present, various precursors have been employed to synthesize high-performance CDs^4–6^, such as the cross-linking/polymerization of biomass, polymers, and organic molecules^7,8^. Among them, o-phenylenediamine (oPD) is one of the most important precursors to CDs due to its self-doping (i.e., N doping) behavior and low cost. oPD molecules have a conjugated structure and serve as both carbon and nitrogen sources, which are the key elements for PL centers^9,10^. Initially, only a variety of blue, green, and yellow emissive CDs with high PLQYs were prepared using oPD as the precursor^11–13^. After the addition of sulfuric acid or nitric acid in the hydrothermal process of oPD, red emissive CDs with fixed PL peak position were successfully obtained^14,15^. Besides strong acids, other additives, such as dopamine had also been incorporated^16^. Despite the large variety of additives, all of the CDs based on oPD show identical red PL emission with fixed peaks at 644 and 680 nm, while their QYs vary greatly. These findings have aroused widespread interest among researchers.

Despite different polymerization processes, large variety, and unclear chemical structures, all the oPD-based CDs have unique constant red PL characteristics (double peak emission). The two current proposed mechanisms of red emission are controversial. The first attributes PL to molecular fluorophores. 3-diaminophenazine fluorophore on the surface of the CDs was considered to decisively change the molecular state of CDs and narrow the photon transition band gap. Through the process of protonation and deprotonation, the change from yellow to red emission was realized^17^. In addition, the formation of quinoxalino [2,3-b] phenazine-2,3-diamine was also proposed to promote the red emission of oPD-based CDs^18^. The second explanation considered the influence of different carbon core structures. The CDs prepared by oPD dimer at higher temperature (250–400 °C) showed a clear size-dependent band gap^19^. At present, an overall investigation on the role of polymer shells, carbon core structures, and their synergistic effect on the red emission of oPD-based CDs is still lacking^20–22^.

To this end, six red emission CDs were synthesized using oPD with/without other chemicals as raw materials, and all of them exhibited the same absorption and PL spectra after purification. The characterization of their structure by a series of tests showed that they had similar carbon core, while spectral characterization confirmed the different surface states of the CDs. Furthermore, transient absorption (TA) spectroscopy combined with single-particle PL spectroscopy technology confirmed that the red emission originated from the transition between different vibrational energy levels in the same PL center. Finally, theoretical calculations combined with thermogravimetric analysis confirmed the formation process of such CDs. Therefore, our work proposes a systematic way to analyze the emission mechanism of oPD-based red emission CDs, which could be used as a guide for the analysis of the structure and mechanism of other types of CDs.

## Results

**Fig. 1:**
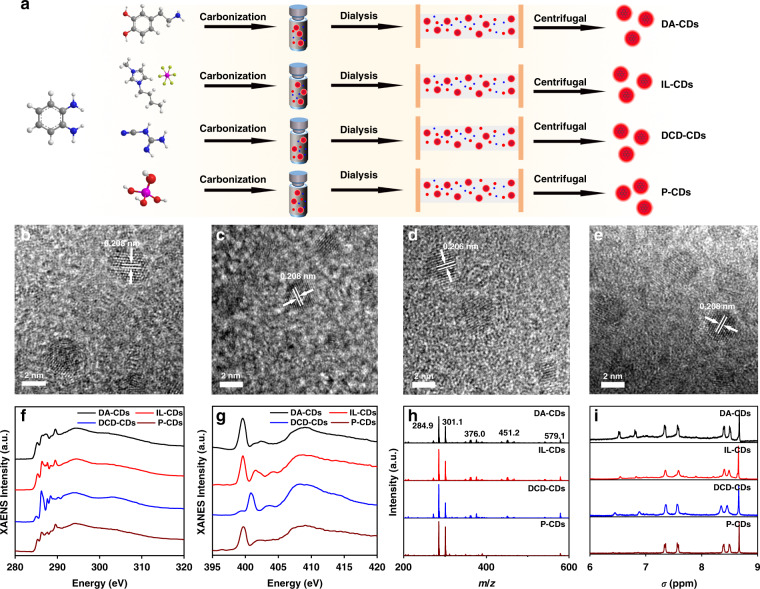
Structural characterization of CDs. Schematic of the fabrication (**a**), HRTEM (**b**–**e**), C (**f**), and N (**g**) *K*-edge XANES spectra, MS (**h**), and ^1^H NMR (**i**) of the four red emission CDs

To better determine the origin of the common red emission spectra of oPD-based CDs, six different synthetic methods were adopted by using oPD as precursors. After dialysis, purification, and centrifugation, six different red emission CDs were obtained. The CDs prepared with oPD and dopamine as precursors were named as DA-CDs. CDs prepared with oPD and ionic liquid (1-butyl-3-methylimidazolium hexafluorophosphate) as precursors were named as IL-CDs, and the CDs prepared with oPD and dicyandiamide as precursors were named as DCD-CDs. In addition to the use of two additional precursors, oPD also resulted in red emissive CDs in the presence of phosphoric acid, sulfuric acid, and nitric acid, which were denoted as P-CDs, S-CDs, and N-CDs, respectively. Since the preparation method and dialysis time were similar, we selected CDs for thin-layer chromatography (TLC) analysis. Using three classical developed mixed solvent ratios (ethyl acetate/petroleum ether, methanol/dichloromethane, ethanol/ethyl acetate), it was difficult to separate the CDs, indicating that our purification was effective (Fig. S2). We selected DA-CDs, IL-CDs, DCD-CDs, and P-CDs as the research objects for subsequent structural and spectral characterization (Fig. 1a). First, the morphology of the prepared CDs is analyzed using transmission electron microscopy (TEM). The results showed that CDs prepared by these methods were uniformly dispersed (Fig. S1), with an average size of 3.75 ± 0.15 nm (Fig. S3). High-resolution TEM (HRTEM) images suggested that the CDs had almost identical lattice fringes, corresponding to the graphene (100) plane (Figs. 1b–e, S4)^23^. As shown in HRTEM, different CDs had different numbers and orientations of the lattice fringes, indicating that the accumulation of carbon nuclei might be different. The X-ray diffraction (XRD) result showed that the CDs had the same broad peak at around 25° (Fig. S5). These results indicated that different preparation methods had almost no effect on the carbon core structure of the CDs. Subsequently, the type of functional groups of CDs was analyzed by FTIR. From Fig. S6, the obtained CDs had the same absorption peaks at 1494, 1641, and 3446 cm^−1^, corresponding to the vibration modes of C-N=, C=O, and -OH/-NH, while the peak at 1055 cm^−1^ belongs to C-O vibration only appeared in CDs prepared under acidic environments^24^. We inferred that the other precursors would react with the C-O on the surfaces of the CDs, connect to the carbon core, and reduce the C-O content. XPS was used to further analyze the surface composition of CDs. Based on the survey spectrum, the CDs contained only three elements (C, N, and O), which were located at 284.8, 398.9, and 531 eV, respectively (Fig. S7)^25^. As anticipated, S-CDs had the lowest N content, while DCD-CDs had the highest N content, which could relate to the different N content in their precursors. The high-resolution C 1s spectrum was divided into three parts at 284.8, 286.1, and 288.5, corresponding to C-C/C=C, C-O/C-N, and C=O. DA-CDs, whose two precursors are both aromatic compounds, had a higher C=C ratio than the other CDs. The introduction of sulfuric acid increased the degree of carbonization on the CDs surfaces, increasing the C=O contents on DA-CDs and S-CDs (Fig. S8). Compared with DCD-CDs, IL-CDs had higher C-O/C-N content (reason?). The high-resolution O 1s spectrum showed that two components (C=O, C-O) on the surface of those CDs were attributed to 531.5 and 533.1 eV. The results of O 1s were very similar to those of C 1s. The addition of sulfuric acid greatly increased the ratio of C=O (Fig. S9). The change in the high-resolution N 1s spectrum was mainly related to its precursor. Some molecules and fragments were connected to the surface of CDs through the formation of covalent bonds, which change the content of aromatic N (398.5 eV) and C-NH_2_ (400.3 eV). From these results, we speculated that the surface structures of these CDs were different, which may affect the optical properties (Fig. S10).

To further determine the possible results of CDs, X-ray absorption near edge structure (XANES) was used to analyze the types of bonds in CDs^26^. XPS, XANES can reflect the entire structure of CDs, the configuration of chemical bonds, and the heterogeneity of the nearest/next neighbor environment in the spectral characteristics. The C K-edge XANES spectra are shown in Fig. 1f. The peak at 294.5 eV corresponded to C 1s core electrons to the δ* symmetric non-dispersion state, and the lowest energy resonance at 285.3 eV corresponded to the C 1s nuclear level electron transition to the Fermi level. The existence of two peaks (285.3 and 294.5 eV) indicated that there were both conjugated and non-conjugated regions in CDs, which further proved the hybrid structure of CDs. These conjugated and non-conjugated structures existed both in the carbon core and a polymer shell. Therefore, we believe that the carbon core and the surface shell of CDs don’t have a clear boundary, but the “density” of the two parts is different. There are more conjugated structures in the carbon core and more polymer chains in the shell. The broad peak at 294.5 eV indicated that the shell structure of these CDs was disordered. The peak at 286.16 eV corresponded to the nearby absorption band related to phenol-C, including N- or O-substituted aryl-C. The peak indicated that some functional groups, e.g. –OH, may exist on the surface of the CDs. DCD had many N-related groups attached to the surface of CDs; therefore, it had a strong absorption peak. Conversely, for P-CDs, the absorption peak was the weakest. The absorption peak near 287.5 eV was attributed to aliphatic-C, while the resonance near 288.4 eV represented the C 1s–π* transition of carboxyl-C. In addition, the σ* peak at 289.5 eV (C 1s → σ*CH transition) was interpreted as an O-alkyl-C group (Fig. 1f). The O K-edge XANES spectra are shown in Fig. S11. The two broad peaks come from the transitions from O 1s to C-O π* (534.7 eV) and C─O σ* (543.2 eV), which are often observed in CD structures decorated with oxygen functional groups^27^. Based on the O *K*-edge XANES, CDs have higher C-O. XANES and XPS spectra comparison suggested that there might be C-O inside the carbon core, that was, different conjugated structures might be connected by C-O. The O *K*-edge XANES and C *K*-edge XANES results were in good agreement. The XANES N *K*-edge spectrum also shows two distinct π* resonances peaks, which are located at 398.5 eV (pyridine N) and 401.2 eV (graphitic N), respectively (Fig. 1g)^28^. The above results indicated that the inside of the carbon core might consist of graphite flakes, and the flakes were connected by bonds, such as hydrogen bonds and C-O. Moreover, the content of pyridine N and graphite N was high in these graphite flakes.

Based on the gel permeation chromatography (GPC) test, each CD had a high molecular weight (MW) and narrow molecular weight distribution^29^. The MW measured by GPC can better reflect the core-shell structure of CDs. The difference in the polymer shell was the key factor leading to the difference in MW. This can also be obtained by XANES. The difference in the XANES spectra was mostly caused by the different linking methods and polymer shell structures. During the hydrothermal reaction, the precursors with polycondensation sites were polymerized/crosslinked under the hydrothermal reactions, whereby some resulting polymers were connected in the shell. For example, there will be more N-substituted aryl-C in DCD-CDs. The MW of P-CDs was the lowest (34.4 kDa), which may relate to the smaller structure of the surface polymer shell (Fig. S12). Furthermore, we used mass spectrometry (MS) to analyze the fragments in the CD structure, and the spectra indicated that these CDs had the same structural fragments. The peak located at 578 Da (molecular ion peak) might correspond to the structure of the graphite flakes accumulated inside the carbon core, while other MS fragment peaks were attributed to the fragments of this large graphite structure. This result agreed well with the XANES spectra (Figs. 1h and S13). From the data, we determined that the carbon core was composed of many conjugated structures that were stacked in parallel and connected by hydrogen, C-O, and C-N bonds. Figure 1i displayed the ^1^H-NMR spectrum. The chemical shifts at 6.5 and 6.6 ppm correspond to the zigzag edge of the polycyclic aromatic hydrocarbons (PAH) structure, while the chemical shift peaks at 7.4 and 7.6 ppm correspond to the phenyl-C structure far away from the amino group. The peaks at 8.1 and 8.5 ppm correspond to the aromatic structure on the armchair side (Fig. 1i and Fig. S14)^30^. Structural characterizations indicated that there was a PAH structure in the CDs, which existed in the carbon core.

**Fig. 2:**
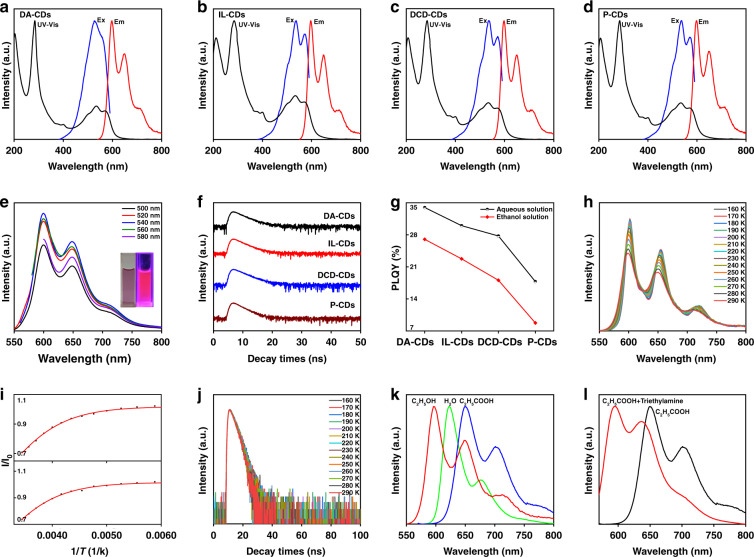
Spectral characterization of CDs. **a**–**d** Em, Ex, and UV-Vis spectra of four CDs in ethanol. **e** Excitation-dependent spectra of P-CDs. **f** PL dynamics of the four selected samples. **g** QYs in aqueous and ethanol solutions. **h**, **i** Variable temperature PL spectra and **j** normalized PL dynamics of P-CDs. **k**, **l** Fluorescence emission spectra of P-CDs in different solvent environments

Next, we further explored the optical properties of the prepared CDs. Except for IL-CDs, all CDs were prepared by hydrothermal methods. The unpurified CDs had characteristic double-peak emission in an aqueous solution, with peaks at 644 nm and 680 nm (Figs. S15 and S16). Then, we performed optical measurements on the purified CDs dispersed in ethanol. The UV-vis absorption peak at 287 nm was due to the π → π* electronic transition of the benzene ring^31^. The absorption peaks located at 500–600 nm were assigned to the C=N absorption of pyridine-N in the carbon core. (Fig. 2a–d). The emission spectrum of CDs in ethanol had two main peaks located at 600 and 650 nm. The peak at 710 nm did not appear in the aqueous solution, which may be caused by the interaction between CDs and the solvent. From the PL spectrum of the purified CDs under different excitation wavelengths, the prepared CDs had excitation-independent bright red emission (Fig. 2e). The steady-state PL lifetime of CDs was measured by a single photon counting method. The four CDs had similar lifetimes in an ethanol solution, which was ~2.6 ns (Fig. 2f). Using the QY test to distinguish the surface polymer shell of these samples, the prepared CDs were found to have higher QYs in an aqueous solution. Among them, DA-CDs had the highest QYs, and P-CDs had the lowest QYs (Fig. 2g). The QYs were 33.96, 29.73, 27.75, and 17.79% in aqueous solution and 27.01, 22.75, 18.08, and 8.76% in EtOH. After purification, the QYs of the CDs decreased, which was mainly due to the departure of some free molecular fluorophores. To understand what fluorescent molecules were outside the dialysis bag, we performed fluorescence analysis on the samples outside the dialysis bag. Three types of dialysate were obtained outside the dialysis bag. The first dialysate was dark blue (D1), the second was yellow (D2), and the third was white (D3). It was not difficult to find that the dialyzed polymer exhibits three luminescent forms, red, cyan, and blue light (Fig. S17). The optical properties of D1 were very similar to those of CDs. Further, we compared D1 and CDs by TLC, and it was not difficult to find that D1 contained some CDs, but also contained a large amount of impurities (Fig. S18). The above conclusions confirmed the necessity of purification, which means removing a series of luminescent molecules and ensuring the purity of the CDs can make the result more reliable. In ethanol solution, the QYs of the four CDs gradually decreased, which might relate to the difference in surface structure. DA-CDs had the largest molecular weight and more surface polymer shells, which promoted the enhancement of QYs. We selected P-CDs as the representative sample for subsequent characterization because P-CDs had the lowest QYs, indicating that they have the most basic structure compared with the other types of CDs. We used temperature-evolved PL spectroscopy to collect the PL spectra of P-CDs in the temperature range of 290–160 K. From Fig. 2h, the PL intensity gradually increased with decreasing temperature. This might be because the non-radiative transition method is related to temperature. The probability of non-radiative transition increased gradually with the temperature. In addition, with the increase in temperature, there was a slight red shift in the PL spectrum. This is because the band gap of CDs decreases with increasing temperature. At low temperatures, the radiation of the surface states of CDs increased. Furthermore, we compared the rate of decrease of the two PL emission peaks in Fig. 2i^32^. The figure shows that the rate of decrease in the two emission peaks was very similar, indicating that the two PL peaks may come from the same PL center (carbon core). Furthermore, the PL intensity of these two peaks was analyzed as a function of temperature as:

1 $$I_{\left( T \right)} = \frac{{I_0}}{{1 + Ae^{ - \frac{{E_{\rm{b}}}}{{k_{\rm{B}}T}}}}}$$

where *k*_B_ is Boltzmann’s constant, *E*_b_ is the exciton binding energy, and *I*_0_ is the integrated emission intensity at 0 K. The *E*_b_ values of the two peaks are similar (152.95 and 144.93 meV). The temperature-dependent lifetime of P-CDs is shown in Fig. 2j. With a decrease in the temperature, the lifetime of the P-CDs gradually becomes longer, possibly because the non-radiative transition method is related to the temperature, which is consistent with the results of the temperature-dependent PL intensity. To further investigate the PL mechanism of these CDs, we conducted a systematic study on the carbon core and surface. As it is well known, NaBH_4_ cannot reduce the benzene ring but can reduce the surface of CDs and the functional groups connected to the carbon core^33^. With the gradual addition of NaBH_4_, the PL emission spectrum of CDs was slightly blue-shifted by 6 nm, indicating that the surface functional groups had a minor effect on the PL emission of CDs (Fig. S19). Then, we added ammonia to the alcohol solution of CDs. Before and after the addition, the color of the solution remained unchanged; thus, the effect of protonation and deprotonation was also excluded (Fig. S20). To further test the effect of protonation and deprotonation on the red PL, we changed the solvent environment to water and changed the pH values. After adding acid to the solution, the color remained unchanged. It can be seen from the PL spectrum that although the acidic environment of the CDs was different, the emission peak was unchanged, which also proves that protonation does not affect the emission wavelength of the CDs. Subsequently, we tested the effect of deprotonation by adding a certain base in the CDs solution, which did not affect the emission wavelength (Fig. S21). Furthermore, we compared the emission spectra of P-CDs in ethanol, water, and acetic acid solutions. The solvent with high polarity promoted red shift in the emission spectrum but did not change the peak shape (Fig. 2k). As mentioned earlier, the acid-base environment did not change the emission wavelength; that is, protonation and deprotonation did not change CD emission. Therefore, the reason for this red-shift phenomenon can be attributed to the solvent effect, which is regulated by the surface shell structure. The presence of the rich polymer shell increased the charge carrier density and induced electron transfer to the edge, resulting in the stable excited state of this structure being strongly influenced by the solvent dipole moment. Then, we added an alkaline substance to the acetic acid solution, and its emission wavelength was blue-shifted, indicating the destruction of its crosslinking structure (Fig. 2l). Finally, to exclude the effect of aggregation, we varied the concentration of the CD solution, and the concentration of H/J aggregate was concentration-dependent. The emission spectra of different concentrations of CDs were tested, and the positions of the emission peaks remain unchanged (Fig. S22). The above results showed that the emission wavelength of CDs is not affected by protonation and deprotonation, and the reason for the wavelength shift is attributed to the dipole-induced electronic state change.

**Fig. 3:**
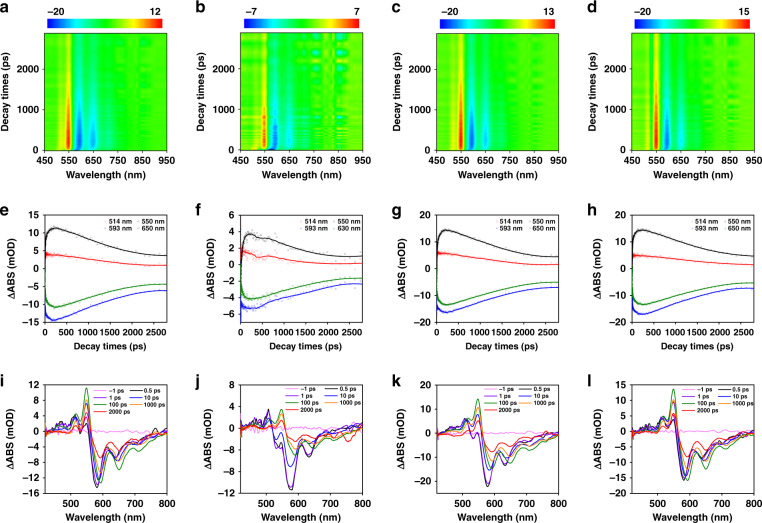
TA and kinetic characterization of CDs. TA spectra of **a** DA-CDs, **b** IL-CDs, **c** DCD-CDs, and **d** P-CDs. Kinetic traces at different probe wavelengths (**e**–**h**). TA spectra of the four CDs at the indicated delay time from 0.5 ps to 2.7 ns (**i**–**l**)

To understand the PL mechanism of oPD-derived CDs, we conducted femtosecond TA measurements with a 400 nm pulsed laser (Fig. 3a–d)^34^. The four samples showed similar results, indicating that they had similar photophysical processes. The negative signal at 580 nm was attributed to transient ground state bleaching (GSB), and the signal at 650 nm corresponded to stimulated emission (SE). The two positive absorption bands at 530 and 550 nm were attributed to excited-state absorption (ESA). The growth of the signal in the initial part was proposed to be the electron relaxation processes from the upper excited state to the lowest excited state via phonon scattering within the electron-phonon coupling in the hybridization carbon core. To clarify the internal energy transfer process, we monitored the carrier relaxation dynamics at different wavelengths after excitation at 400 nm, as shown in Fig. 3e–h^35^. The evolution of the entire spectrum with different time delays of different samples (Fig. 3i–l) is also depicted. The decay kinetic curve of GSB was fitted with a tri-exponential function. Furthermore, a global fit to the TA data was used to obtain the time scale of different processes. The fitted lifetimes of carriers in DA-CDs were 3.34 ± 0.05, 80.76 ± 0.92, and 2383.66 ± 9.47 ps. The changes in the spectra at different lifetimes were obtained from decay-dependent difference spectroscopy (DADS), as shown in Fig. S17a. The three DADS values of the four samples were also almost the same, which proves that the photophysical processes are the same for all four CDs, consistent with the previous results (Fig. S23). After excitation at 400 nm, the remaining hot carriers in the carbon core were scattered by optical phonons (3.34 ps) and acoustic phonons (80.76 ps) to the lowest excited state that emitted luminescence via electron–hole recombination within 2.38 ns. The fitting results of the remaining CDs were similar, and the carriers went through the same relaxation channel (Figs. 3e–g and S24b–d)^36^.

**Fig. 4:**
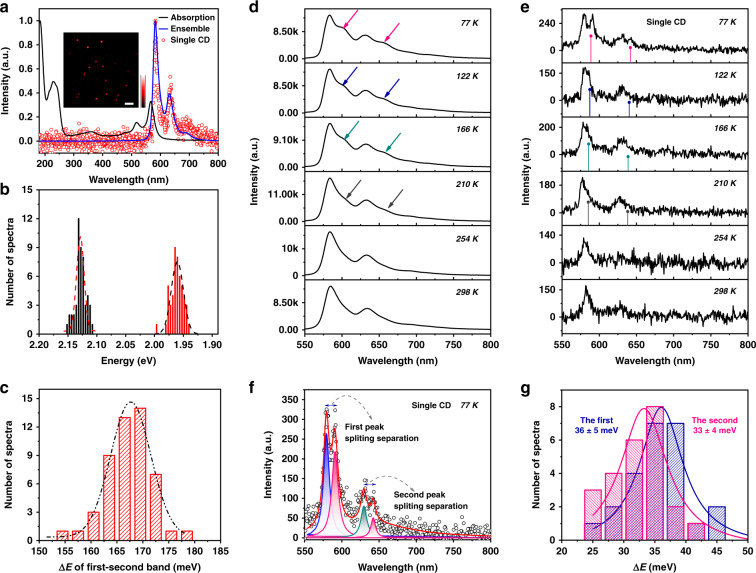
Optical properties of ensemble and single-particle CDs. **a** Normalized absorption (black) and photoluminescence (PL) spectra of ensemble (blue) CDs and the representative single-particle CD (red) PL spectra. **b**, **c** are the distributions of the maximum emission peak and spectral band separation, respectively. The dotted curves in **b**, **c** are the distributions fitted with Gaussian functions; fluorescence images of single-particle CDs placed on a PVA film (inset). PL spectra of the ensemble CDs (**d**) and the single-particle CDs (**e**) at different temperatures from 77 to 298 K. **f** PL spectra of single R-CDs at 77 K. Black circles represent the measured data; red curves correspond to the total fits that contain four Lorentzian functions. **g** Distributions of the spectral band separation of dozens of single-particle CDs at 77 K

The above results indicated that the oPD-derived CDs were similar, while the surface polymer shell layer and the carbon core were stacked in different ways, which may affect the PL properties. To further determine the contribution of the carbon core and the polymer shell on the CD’s PL spectra, single particle spectroscopy was employed. It is universally acknowledged that the emissive impurities and defects in the slide influence the precision of measurements. Therefore, we selected PVA as the matrix, and CDs were diluted and spin-coated on a PVA-covered cleaned slide. Uniform bright spots in wide-field imaging represent monodispersed CDs (inset of Fig. 4a). The normalized PL and absorbance spectra of the ensemble and individual CDs are shown in Fig. 4a. The absorption peaks at 420–700 nm were mirrored and symmetrical with the emission spectrum, which is characteristic of vibrionic overtone bands. The local matrix environment resulted in a slight blue shift in the CDs’ PL spectra^37^.

In addition, the asymmetric PL spectra of individual CDs indicated that several vibrionic transitions were involved in the relaxation process. All the spectra of individual CDs were highly consistent with the bulk spectrum. Next, we fitted the PL spectra of single CDs and recorded the maxima. The distribution of the emission peaks of 50 individual CDs is shown in Fig. 4b, c. The width of the main emission peak distribution was ~43 meV (~15 nm), which was narrower than that of the reported single CD at ~180–480 meV (~40–120 nm)^38,39^. Statistical analysis of peak positions and spectral band separation in single-particle spectra revealed that the single CD PL was nearly identical to that of the CDs at the ensemble level, with well-resolved vibrionic structures, and it was consistent with nearly monodisperse optical properties^40^. Furthermore, we utilized temperature-dependent PL to gain insights into the origin and mechanism of CDs using a custom-built wide-field microscope, as shown in Fig. 4d. These samples were excited with 532 nm continuous laser light. Notably, the PL spectra of the CDs showed distinct asymmetry of each vibrionic structure at temperatures lower than 210 K, which resembled spectral splitting. A side-peak emission was spectrally separated from each vibrionic overtone band at low temperature, which was located at lower energy. The anomalous phenomena of emission peak splitting is frequently found with semiconductor QDs, organometallic halide perovskite, and other two-dimensional particles but not in carbon nanomaterials due to crystal structure transitions or the coupling between thermal carriers^41,42^.

To test whether collective effects are responsible for the spectral splitting of the CD film, we collected the emission spectra of an individual CD at different temperatures, as shown in Fig. 4e. At 77 K, two distinct splitting peaks were visible at ~591 and 642 nm, but the weak intensity did not allow us to analyze the third peak splitting at ~685 nm. The two main splitting peaks broadened and began to overlap when the temperature was increased to 210 K. At 298 K, two broad featureless peaks were observed, which engulfed the spectroscopic structure observed at lower temperatures. The observation of splitting peak emission from single-particle CD for the same wavelength range indicated that the splitting emission feature is not due to CD stacking. While the relative amplitudes of the splitting features vary with temperature, we observed that their strength decreased rapidly with temperature increasing. The variation in peak splitting at different temperatures indicated that the emission could be modulated by the electron-phonon coupling between the chromophore and the longitudinal optical modes^43–45^. To further determine the mechanism of spectral splitting, we calculated the energy splitting of the two bands by fitting the ensemble and single CD spectra, as shown in Fig. 4f. Statistical analysis of the separation of splitting peaks revealed another interesting aspect of CD photophysics. The distribution of the energy difference of the main peaks and shoulder peaks plotted in Fig. 4g had almost the same value, differing by only a few meV. This could be attributed to electron−phonon coupling. The generation of the high-energy main band was due to the excitation of quantum vibrations and zero-phonon transitions in the graphene structure. Significantly, electron−phonon coupling occurs between the delocalized phonons of the carbon core and the chromophores^38^.

**Fig. 5:**
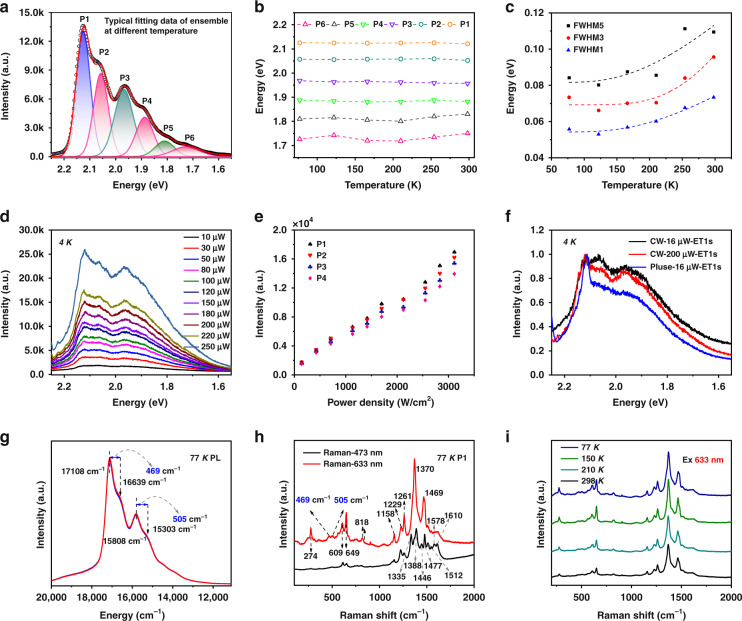
Temperature dependence of the PL properties of the CDs. **a** PL spectrum at 77 K fitted by the sum of six Gaussian peaks, with separate P1 to P6 components. The phonon sidebands are shown in pink. **b** Emission peak energies and **c** the line widths of P1, P3, and P5 as a function of temperature. **d** PL spectra at 4 K with different excitation intensities. **e** PL intensity as a function of power density. **f** Comparison of the PL spectra of pulsed and continuous light excitation. **g** PL spectrum of R-CDs with the abscissa as the wavenumber. **h** Raman spectra of R-CDs excited at 473 and 633 nm at 77 K. **i** Raman spectra of CDs at 633 nm at different temperatures from 77 to 298 K

To better understand the PL response mechanism, the PL spectra under different temperatures were fitted using multi-Gaussian functions. The band gap, bandwidth, and energy difference between vibrionic overtone bands and phonon sidebands were investigated, as shown in Fig. 5a, b. It is worth noting that these four bands (P1–P4) remained almost unchanged, with slight fluctuations of several meV, which were closely correlated with the conformation. Unlike conventional QDs, CDs undergo more complex synthesis stages, including polymerization and carbonization. Due to the uncertainty of aggregation, the structure is promoted to expand in all directions, which increases the conformational disorder. Further carbonization enhanced the stability and rigidity of CDs. Therefore, the excitons created in CDs by photo-excitation will be confined in a locally conjugated region, reducing the effect of temperature on the electronic state.

The FWHM of each dominant peak was also investigated, as shown in Fig. 5c. Generally, the majority of CDs obtained by the bottom–up route possess broad PL spectra, which is difficult to explain by size polydispersity alone. Inhomogeneous and homogeneous broadening components should be considered together for this complex system. In principle, the time of electron relaxation is related to the spectral broadening of the PL bandwidth, as expressed in the following equation:

2 $$b\Gamma = \frac{1}{{2\pi c}} \times \frac{1}{{T_2}} = \frac{1}{{2\pi c}}\left( {\frac{1}{{2T_1}}} \right. + \left. {\frac{1}{{T_2^ \ast }}} \right)$$

where *T*_1_ stands for the population relaxation time including both radiative and nonradiative processes; *T*_2_ is the total dephasing times; and $T_2^ \ast$ is the pure dephasing time^46^. Inhomogeneous broadening is independent of temperature, mainly due to changes in the shape, size, and composition of CDs. In contrast, homogeneous broadening originates mainly from exciton scattering by acoustic and optical phonons, where acoustic phonons dominate at lower temperatures and optical phonons contribute at higher temperatures. The overall broadening can be simplified as follows:

3 $${{{\mathrm{FWHM}}}}\left( T \right)\;{{{\mathrm{= }}}}\;\frac{A}{{{{{\mathrm{exp}}}}\left( {\frac{{E_{{{{\mathrm{ph}}}}}}}{{k_{{{\mathrm{B}}}}T}}} \right) - {{{\mathrm{1}}}}}}{{ \;+ \;{C}}}$$

where *A* is the exciton–longitudinal optical phonon (LO) coupling strength, *E*_ph_ represents the LO phonon energy, and *C* is the inhomogeneous broadening term. The curves of FWHM fit well using this formula. The FWHM of the three main peaks increased slowly with increasing temperature, which showed the narrowed FWHM might come from the reduced LO phonon coupling. The LO phonon energy strengths were 59, 79, and 65 meV, and the coupling strengths were 0.29, 0.53, and 0.23, respectively. Furthermore, we investigated the PL spectrum of CDs at 4 K, and the side peaks became more pronounced than at 77 K (Fig. 5d). Figure 5e shows the measured PL intensity as a function of excitation power density for P1–P4. The PL intensity of P1–P4 increased linearly with excitation power density. This behavior suggested that there is no biexcitonic origin of the low energy line, in which case the evolution would be quadratic. In contrast, a sharp emission peak was observed under pulsed laser excitation, which would contribute to amplified spontaneous emission (ASE) from CDs. These results also ruled out ASE as the cause of side peaks. Based on the results in Figs. 4d–g and 5a–f, we assigned the main emission peaks (P1 and P3) to the vibrionic overtone bands and the side peaks in the low-energy tail to the phonon-side band of the transition. However, it is still unclear whether the origin of the vibration coupled to the electronic transition comes from the nuclear motion of the matrix or the phonon modes of the CD backbone^47–49^.

To understand the origin of the vibration coupled to the electronic transition, distributions of the mean phonon energies were further studied. The energy difference of the spectral band separation was calculated and translated into wavenumbers 469 and 505 cm^−1^ (Fig. 5g). Raman spectra were measured to identify features of phonon coupling in spectral splitting (Fig. 5h). By changing the excitation light source to balance the PL interference, the resonant Raman signal of a high signal-to-noise ratio was obtained. The obvious signature D (~1370 cm^−1^) and faint G (~1580 cm^−1^) bands appeared under 633 nm excitation, corresponding to the disordered structures and weak translational symmetry of the CDs, respectively. Several sharp transitions were attributed to C=N, C–N, and N–H bending which are observed at 1469, 1370, and 1229 cm^−1^. We further compared the Raman spectra of the other three CDs and o-phenylenediamine, and the results are shown in Fig. S25. The Raman spectra of the four CDs are almost the same but clearly different from o-phenylenediamine, indicating that the characteristic signals mainly come from the CDs rather than the residual o-phenylenediamine or other small molecules. These Raman spectra of oPD-based CDs had more fine Raman modes, which was different from typical graphite or GQDs. This may be due to the cross-linking of many unknown molecule-like polymer chains on the surface of the CDs^50–52^. More importantly, the frequency of the coherent vibration matched that of the phonon of CDs (the energy difference of the spectral band separation), indicating that the vibration coupled to the electronic transition comes from the CDs backbone. The corresponding characteristic peaks were not observed in the reference sample, eliminating the possibility that the vibration coupled to the electronic transition comes from the nuclear motion of the matrix. These results directly prove that CDs are new materials, different from conventional quantum dots (QDs) and organic molecules, but possess both of their properties. Furthermore, the Raman peak position of CDs was independent of temperature, indicating the conformational disorder (rigid structure) of CDs. More succinctly, the temperature independence of the PL peak position was attributed to the rigid structure of oPD-derived CDs, which limits π-electron delocalization and exciton migration. The spectral splitting originates from the coupling of the electronic transition and vibration of the carbon core.

**Fig. 6:**
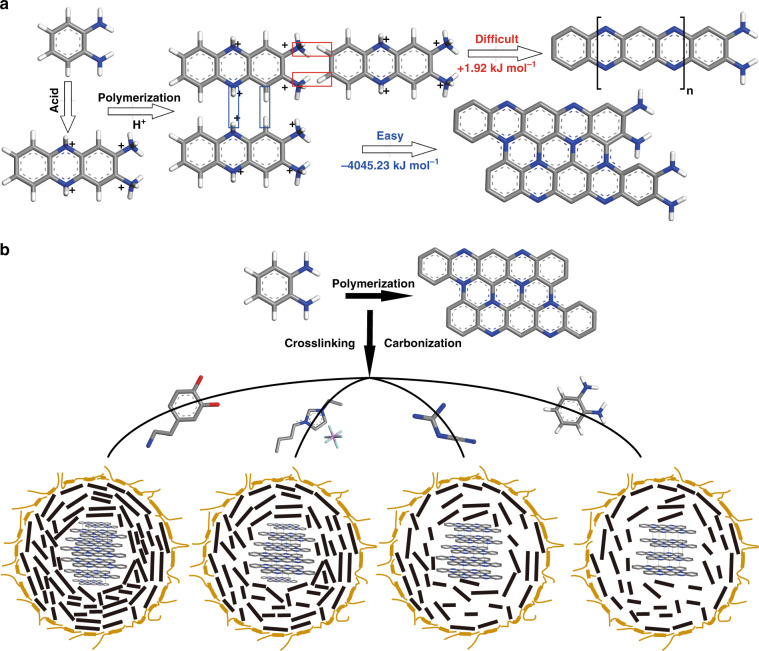
Formation process and structural analysis of CDs. **a** Formation energies of longitudinal and transverse growth of CDs from oPD. **b** Core–shell structures formed by four kinds of CDs

The analysis presented here proved that CDs had a core-shell structure and a unique form between QDs and organic molecules. To further investigate the formation process and structure of CDs, we conducted a comparative experiment. Linear poly-oPD was prepared by the method commonly used in the literature for comparison^53^. As shown in Fig. S26a, the ^1^H NMR spectrum of the oPD polymer (PoPD) obtained by oxidation of (NH_4_)_2_S_2_O_8_ showed a strong peak at 6.3 ppm. The double peak may be related to the protonation of -NH_2_. The peak at 6.6 ppm was attributed to aromatic protons. In addition, there are two broad, weak peaks at 7.4 and 7.6 ppm, which may be due to the protons on the 1, 2, 4, and 5-tetrasubstituted benzene rings (Fig. S26b). The oPD also showed a characteristic MS spectrum, which was mainly dissociated into monomers, dimers, trimers, and tetramers under high voltage. Both CDs and polyanilines had similar conjugated structures, which might make them look similar in ^1^H NMR, but their MS was quite different. Additionally, the spectral data and surface functional groups were different. Then, we performed optical measurements on the purified PoPD dispersed in ethanol. The emission spectrum of PoPD in ethanol had a main peak located at 411 nm, indicating the presence of quinoid imine units (-C=N-)^54^. From the PL spectrum of the purified sample under different excitation wavelengths, the prepared PoPD had excitation-independent yellow emission (Fig. S27). Furthermore, to make the PL mechanism robust, we compared the PL spectra of CDs, 2,3 diaminophenazine (DPA), PoPD, and 2,3 diaminophenazine-based CDs by hydrothermal treatment with (DPA-CDs-S) and without H_2_SO_4_ (DPA-CDs) (Figs. S28–S32). As can be seen from Fig. S28, the optical properties of DPA were similar to those of PoPDs but different from those of CDs. When DPA was hydrothermally reacted in two environments, DPA-CDs (without H_2_SO_4_) showed yellow light emission similar to PoPD, while DPA-CDs-S (with H_2_SO_4_) had optical properties similar to CDs. To better compare with CDs, we dispersed these materials in different concentrations of H_2_SO_4_. The total volume of the test solution was maintained at 3 mL, and the fluorescence change after adding 50–500 μL H_2_SO_4_ was compared. For PoPD, the fluorescence emission peak did not change with the higher concentration of H_2_SO_4_, but the fluorescence intensity gradually weakened, and the position of its absorption peak remained unchanged (Fig. S29). For DPA, its optical properties were similar to those of PoPD because the structure was simple. And thus with the addition of H_2_SO_4_, the fluorescence intensity of DPA decreases rapidly. When 500 µL of H_2_SO_4_ was added, the fluorescence was nearly quenched, but a shift in the fluorescence emission and absorption peaks was not observed (Fig. S30). After the hydrothermal oxidation of DPA to DPA-CDs, the absorption spectrum did not change completely compared to PoPD and DPA, which proved that the formation of carbon dots increases the stability of the system (Fig. S31). Finally, we added sulfuric acid during the hydrothermal reaction of DPA, and the prepared DPA-CDs-S exhibited a characteristic double-peak emission with an absorption peak at 620 nm, and its fluorescence emission wavelength was not affected by H_2_SO_4_ (Fig. S32). The above structure further confirmed our proposed CD formation process: oPD first formed DPA, and then DPA tended to grow vertically in an acidic environment. The results showed that CDs were distinct from these structures. In addition, the CDs prepared from DPA also exhibited characteristic double peak emission, which proved that the formation process of CDs in an acidic environment may form DPA first. Subsequently, to determine whether the functional group compositions of PoPD and CDs were similar, structural analysis was performed by XPS and FTIR. From the XPS survey spectrum, it could be seen that PoPD only contains four elements (C, N, O, and S) (Fig. S34). It was not difficult to find that PoPD contained S, which might be related to the preparation method. Due to an oxidation process in its preparation, PoPD contained a large amount of O. It could be seen from the N 1 s spectrum that the existence of the linear structure made PoPD contain a large number of =N- and -NH- groups. From Fig. S33, the peak between 3380–3145 cm^−1^ in the PoPD FTIR spectrum was assigned to -OH/-NH, and the strong absorption peak at 1574 cm^−1^ was assigned to the C=N vibration, which indicated that there were more imine and phenazine structures in PoPD. The bands at 750 and 601 cm^−1^ were characteristic peaks of out-of-plane bending vibrations of aromatic benzene with a phenazine skeleton^55^. The main absorption peaks of PoPD and CDs were quite different. Therefore, it could be seen from the spectral and structural characterization that PoPD and CDs were two completely different structures with different fluorescence emission, functional groups, and configurations. Subsequently, the polymer characteristics and chemical structure of CDs and PoPD were also characterized by derivative thermogravimetric analysis (DTG) and thermogravimetric analysis (TGA) (Fig. S35). The two materials showed similar TGA curves. After heating to 800 °C, 50% and 52% of CDs and PoPD remained, respectively. However, as shown in the DTG curve, the heat absorption and release processes of the two were completely different^56^. PoPD had three stages of thermal transition. The thermal transition before 200 °C was mostly related to the water attached to the surface. The second thermal transition between 240 and 320 °C, was attributed to the loss of oligomers, and the last thermal transition (320–700 °C) corresponded to the degradation of the backbone^57^. In comparison, only one major thermal change and several minor changes were observed in the thermogravimetric data of CDs, which was different from the linear polymer process. This proved that the prepared CDs were not linearly polymerized. The main thermal change corresponded to the cracking of the carbon core, while the previous slight change may correspond to the decomposition of the surface shell. To further determine the polymerization process and the polymerization products of CDs, the polymerization method of oPD was analyzed by density functional theory calculation^58,59^. After acid-assisted protonation, polymerization can be carried out in two ways: longitudinal (formation of wide conjugated planar fragments) and lateral growth (formation of long linear polymer chains). The lateral growth (+1.92 kJ mol^−1^) required much higher energies than longitudinal growth (−4045.23 kJ mol^−1^), based on the calculated formation energies. The results showed that oPD tends to aggregate and formed planar structures. Then, the planar structures self-assembled into spherical CDs (Fig. 6a). Through the previous characterization, we could roughly infer that these four CDs had similar carbon core structures, but the stacking form inside the carbon core was different from the surface polymer shell and polymer chains. CDs with high QYs may have a denser carbon core structure and flexible polymer shell with more surface polymer chains (Fig. 6b).

## Discussion

This work proposed a merged PL mechanism to resolve the debate regarding the PL origin of oPD-based red emission CDs. Six red emission CDs based on oPD precursors were obtained through six different preparation methods. These six CDs had similar PL behaviors, and it was preliminarily inferred that their PL sources were the same. The detailed structural characterization of the material further confirmed this hypothesis and indicated that the conjugated structure within the carbon core affected its emission wavelength. Moreover, the electronic interaction between the surface polymer shell and carbon core adjusted the QYs of CDs. TA spectroscopy combined with single-molecule PL spectroscopy confirmed that the PL double-peak emission of these CDs originated from the transition of different vibrational energy levels in the same PL center, that is, the electronic structure of the carbon core. Finally, theoretical calculations were used to reveal the formation process of such CDs. We believe this article clarifies the general PL mechanism and formation process of oPD-based red emission CDs. This unified mechanism provides a new method for analyzing the structure-property relationship of CDs and thus opens a new way for analyzing other types of CDs, thereby uncovering untapped opportunities.

## Supplementary information

SUPPLEMENTAL MATERIAL
