# Supplementary material for: Electron–phonon coupling-assisted universal red luminescence of o-phenylenediamine-based carbon dots
Source: Light Sci Appl. 2022 Jun 6;11:172. doi: 10.1038/s41377-022-00865-x (PMC9170735; doi:10.1038/s41377-022-00865-x)
Supplement: Supplementary file 1 — SUPPLEMENTAL MATERIAL [file 41377_2022_865_MOESM1_ESM.docx]

Supplementary Information for

Electron-Phonon Coupling Assisted Universal Red Luminescence of o-phenylenediamine-based Carbon Dots

Boyang Wang^a,#^, Zhihong Wei^b,#^, Laizhi Sui^c,#^, Jingkun Yu^a^, Baowei Zhang^d^, Xiaoyong Wang^e^, Shengnan Feng^e^, Haoqiang Song^a^, Xue Yong^f^, Yuxi Tian^b^*, Bai Yang^g^, Siyu Lu^a^*

^a^ Green Catalysis Center, and College of Chemistry, Zhengzhou University, Zhengzhou 450000, China. Email: sylu2013@zzu.edu.cn. Tel: +86 13598052106

^b^ Key Laboratory of Mesoscopic Chemistry of MOE, School of Chemistry and Chemical Engineering, Jiangsu Key Laboratory of Vehicle Emissions Control, Nanjing University, Nanjing 210023, China. Email: Email: tyx@nju.edu.cn. Tel: +86 17712400641

^c^ State Key Lab of Molecular Reaction Dynamics, Dalian Institute of Chemical Physics, Chinese Academy of Sciences, Dalian, 116023, China.

^d^ Nanochemistry Department, Istituto Italiano di Tecnologia (IIT), via Morego 30, 16163 Genova, Italy.

^e^ School of Physics, National Laboratory of Solid State Microstructures, Collaborative Innovation Center of Advanced Microstructures, Nanjing University, Nanjing 210093, China.

^f^ Department of Physics and Engineering Physics, University of Saskatchewan Saskatoon, S7N5E2, Canada.

^g^ State Key Lab of Supramolecular Structure and Materials, College of Chemistry, Jilin University,

Changchun 130012, China

^#^ B. W., Z. W., and L. S. contributed equally.

**Table of Content**

1. **Experimental Procedures.**
2. **Figure S1.** Separation of CDs in different developing agents.
3. **Figure S2.** TEM imaging of six CDs.
4. **Figure S3.** Size distribution of six CDs.
5. **Figure S4.** HRTEM of S-CDs and N-CDs.
6. **Figure S5.** XRD patterns of six CDs.
7. **Figure S6.** FTIR of six CDs.
8. **Figure S7.** XPS spectra and element ratios of six CDs.
9. **Figure S8.** C1s spectra and ratio of six CDs.
10. **Figure S9.** O1s spectra and ratio of six CDs.
11. **Figure S10.** N1s spectra and ratio of six CDs.
12. **Figure S11.** N K-edge absorption spectra of four selected CDs.
13. **Figure S12.** Molecular weight distributions of four selected CDs.
14. **Figure S13.** Mass spectrometry analysis of S-CDs and N-CDs.
15. **Figure S14.** ^1^H NMR analysis of S-CDs and N-CDs.
16. **Figure S15.** Aqueous fluorescence spectra of six CDs.
17. **Figure S16.** Fluorescence excitation-dependent spectroscopy in aqueous solution of P-CDs.
18. **Figure S17.** PL and UV–Vis spectra of D1, D2, and D3.
19. **Figure S18.** Separation of D1 and CDs by TLC.
20. **Figure S19.** PL spectra of the P-CDs in different concentrations of NaBH_4_.
21. **Figure S20.** PL spectra of the P-CDs in different alkaline environments in EtOH.
22. **Figure S21.** PL spectra of the P-CDs in different acid/alkaline environments in aqueous solution.
23. **Figure S22.** PL spectra of the P-CDs in different concentrations in EtOH.
24. **Figure S23.** Normalization of the third DADS of four selected CDs.
25. **Figure S24.** DADS spectra of four selected CDs.
26. **Figure S25.** Raman spectra of three other CDs and oPD.
27. **Figure S26.** ^1^HNMR and MS of PoPD.
28. **Figure S27.** PL and UV–Vis spectra of PoPD.
29. **Figure S28.** PL spectra of PoPD, DPA, DPA-CDs and DPA-CDs-S.
30. **Figure S29.** PL spectra of PoPD with different concentrations of H_2_SO_4_.
31. **Figure S30.** PL spectra of DPA with different concentrations of H_2_SO_4_.
32. **Figure S31.** PL spectra of DPA-CDs with different concentrations of H_2_SO_4_.
33. **Figure S32.** PL spectra of DPA-CDs-S with different concentrations of H_2_SO_4_.
34. **Figure S33.** XPS spectra of PoPD.
35. **Figure S34.** FTIR comparison of four selected CDs and PoPD.
36. **Figure S35.** DTG and TGA of CDs and PoPD.

**Experimental Procedures**

**Materials:** O-phenylenediamine (oPD), Dopamine, (1-butyl-3-methylimidazolium hexafluorophosphate, Dicyandiamide, H_2_SO_4_, HNO_3_, and HCl were purchased from Sigma-Aldrich Co. LLC. All of the chemicals were used directly without further purification. The redistilled water used in this experiment was purified via the SZ-93A water purification system. All the reagents were analytical grade and utilized without further purification.

**Preparation of 6 kinds of Carbon Dots (CDs)**:

The 6 kinds of red emission CDs were synthesized by using oPD as one of the precursors at the same temperature and reaction time.

DA-CDs: oPD (108 mg), Dopamine (184 mg), 10 mL of redistilled water, and 1 mL of H_2_SO_4_ were added to a 25 mL autoclave and heated at 200°C for 6 h.

IL-CDs: oPD (108 mg), (1-butyl-3-methylimidazolium hexafluorophosphate (450 mg), and 10 mL of ethanol were added to a 25 mL autoclave and heated at 200°C for 6 h.

DCD-CDs: oPD (108 mg), Dicyandiamide (84 mg), 10 mL of redistilled water, and 1 mL of H_2_SO_4_ were added to a 25 mL autoclave and heated at 200°C for 6 h.

P-CDs: oPD (108 mg), 10 mL of redistilled water, and 1 mL H_3_PO_4_ were added to a 25 mL autoclave and heated at 200°C for 6 h.

S-CDs: oPD (108 mg), 10 mL of redistilled water, and 1 mL H_2_SO_4_ were added to a 25 mL autoclave and heated at 200°C for 6 h.

N-CDs: oPD (108 mg), 10 mL of redistilled water, and 100 μL HNO_3_ were added to a 25 mL autoclave and heated at 200°C for 6 h.

After cooling to room temperature, the solution was centrifuged at 10000 rpm for 5 min, filtered through a 0.22 μm filter membrane, and finally dialyzed with the membrane (1000 Da) for 7 days to remove the unreacted precursor and acid. The liquid in the dialysis bag was centrifuged, and the precipitate was washed several times and then freeze-dried.

**Characterization of CDs:** Transmission electron microscopy (TEM) images were acquired with an FEI TECNAIG2F20-S-TWIN electron microscope. X-ray photoelectron spectroscopy (XPS) measurements were performed using a Thermo Fisher ESCALAB 250Xi surface analysis system. X-ray diffraction (XRD) patterns were obtained using an X-ray diffractometer (PANalytical, X’Pert PRO). The absorption and fluorescence spectra of the CDs were recorded on a Persee TU-1810PC spectrophotometer and FLS1000 fluorescence spectrophotometer at room temperature, respectively. Fourier transform infrared spectroscopy (FTIR) was performed on a Nexus 470 (Thermo Fisher) spectrometer.

**Femtosecond transient absorption setup:** A regeneratively amplified Ti: sapphire laser system (Coherent Libra, 50 fs, 1 kHz) provides the fundamental light source. The pump pulse (400 nm) is generated by focusing a portion of the fundamental light into the BBO crystal. To avoid the influence of rotational relaxation effects on dynamics, the polarization of the pump pulse is randomized by a depolarizing plate. The other fundamental pulse provides a broadband probe pulse (white light continuum) that is produced by focusing 800 nm of fundamental light into the sapphire plate (3 mm). The pump and probe beams are overlapped in the sample with crossing areas of 600 μm and 150 μm. After passing through the sample, the probe pulse is focused into an optical fiber coupled to a spectrometer (AvaSpec-1650F). The energy of the 400 nm excitation pulse is adjusted to about 1.5 μJ/pulse by a neutral density optical filter. The pump pulse is chopped at 500 Hz to acquire pumped (signal) and un-pumped (reference) probe spectra, and the ∆OD spectrum can be obtained by processing them. The solutions were placed in a 2 mm optical path length quartz cuvette. Both the instrument response function (100 fs) and temporal chirp in the probe light are determined by measuring the cross-modulation of ethanol. The group velocity dispersion effect on the experimental data is corrected by a homemade chirp program. For each measurement, the pump-probe delay scan is repeated three times to give the averaged experimental data.

**Single-particle spectroscopy:** The spectral data of the CDs were analyzed using a home-built, widefield microscope. A 532-nm continuous-wave laser diode with a laser power density of 48 W/cm^2^ on the sample surface was used as the excitation source. The fluorescence of the CDs under different conditions was obtained using an oil immersion objective lens (Olympus UPlanFLN 60×, NA = 1.25) and detected using an EMCCD camera (iXon Ultra 888, Andor) after being passed through a 550-nm long-pass filter (ET550lp, Chroma). A transmission grating (Newport, 150 lines per mm) was placed in front of the camera to obtain the fluorescence spectra. PL lifetime measurements were performed using a single-photon counting system (TCSPC, PicoHarp 300) with 532-nm excitation light from a super continuous laser (Fianium SC-400, 40 MHz).

**Temperature-dependent Raman spectra**: Temperature-dependent Raman spectra were measured by a micro-Raman spectrometer (Horiba-JY HR Evolution) with a cw laser operating at 532 nm and 633 nm. The signal was acquired through a ×100 objective (NA=0.85, Leica) and 600 lines per millimeter grating. A cryostat (Cryo Industry of America) was used to provide a vacuum environment and temperature control from 77 to 300 K by liquid nitrogen flow.

**Computational details:** All calculations are performed with the Gaussian 09 program. The ground-state geometries of carbon dots were optimized by DFT B3LYP (Becke's three-parameter hybrid function with the non-local correlation of Lee-Yang-Parr) functional, 6-31G(d) basis set (B3LYP/6-31G(d). The absorption spectra of carbon dots were calculated using the TDDFT method (at the B3LYP/6-31G(d) level) based on optimized ground-state geometries. The first excited state was optimized using the TDDFT method to calculate the emission energy (wavelength) which is the energy difference between the ground and the first excited state.

**Results and Discussion**


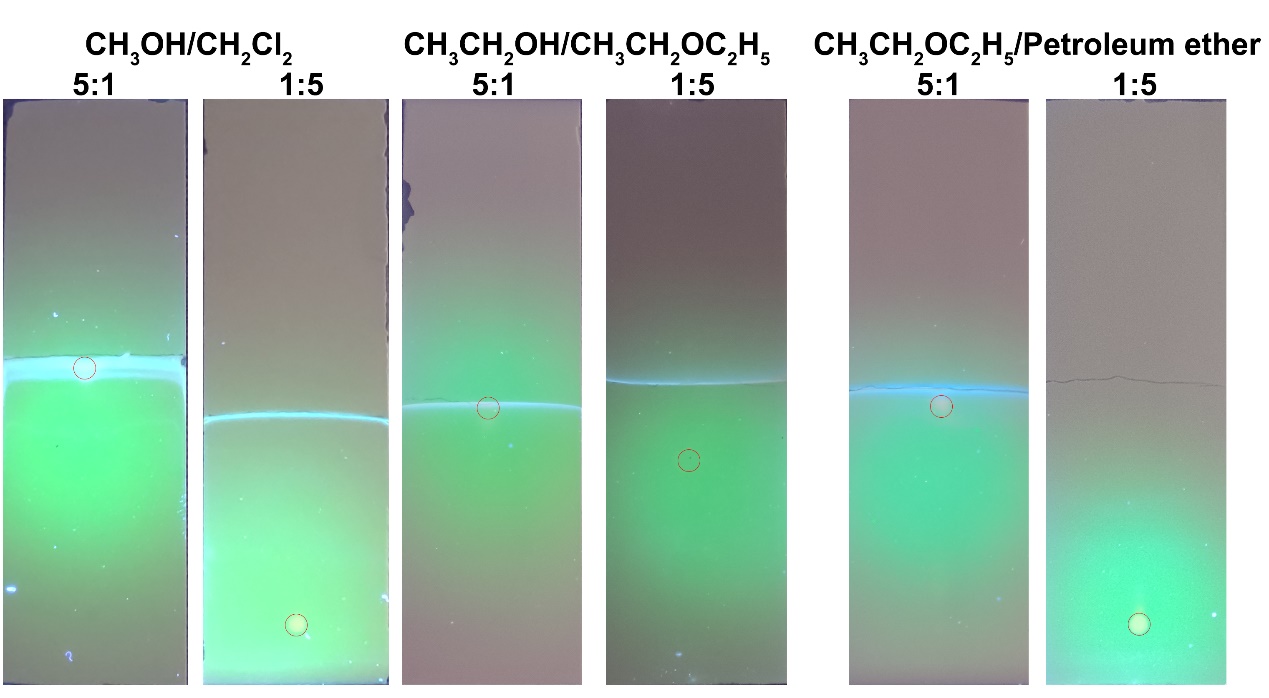


**Figure S1.** Separation of CDs in different developing agents.


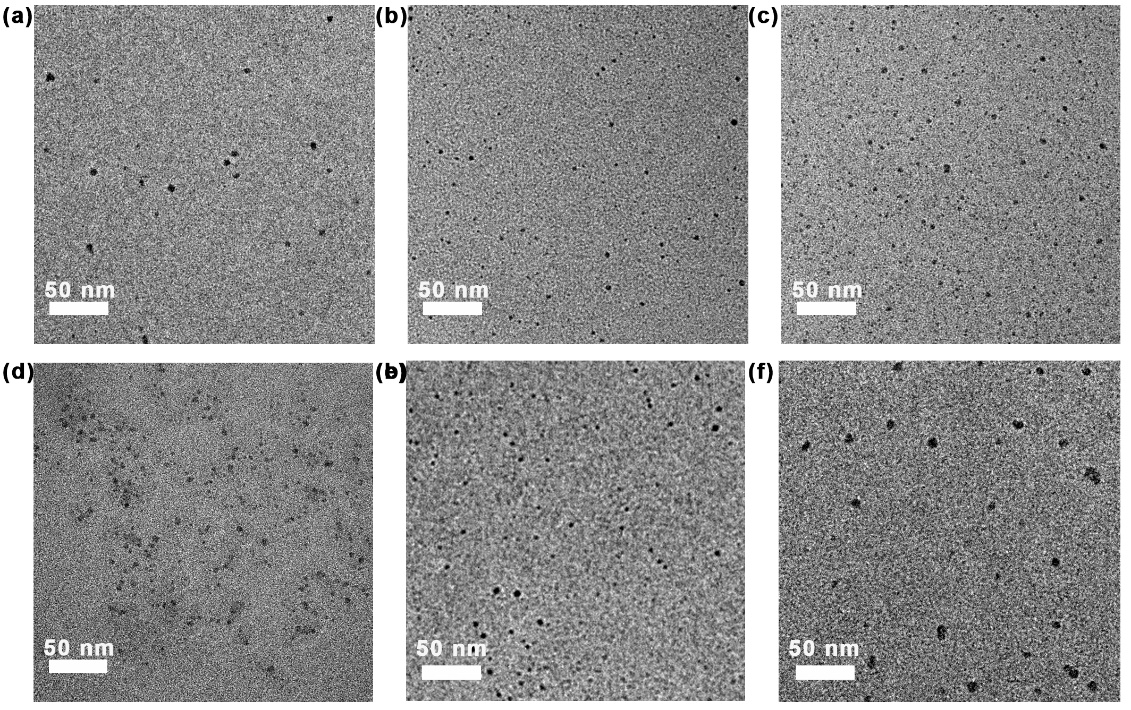


**Figure S2**. TEM imaging of DA-CDs (a), IL-CDs (b), DCD-CDs (c), P-CDs (d), S-CDs (e), and N-CDs (e).


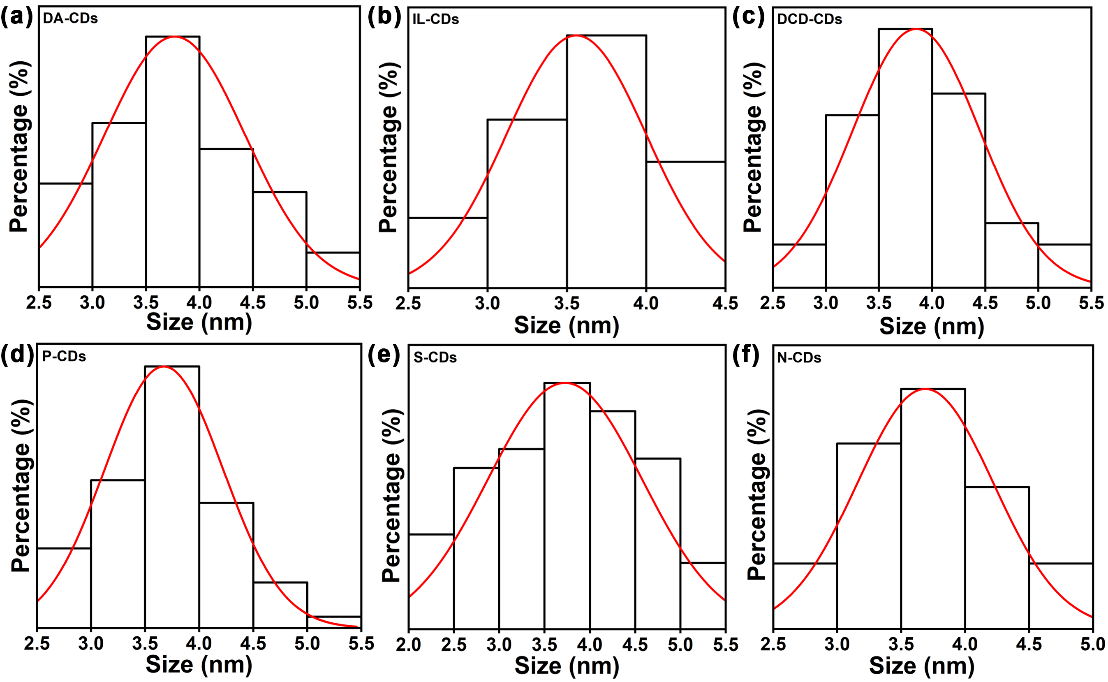


**Figure S3.** Size distribution of DA-CDs (a), IL-CDs (b), DCD-CDs (c), P-CDs (d), S-CDs (e), and N-CDs (e).


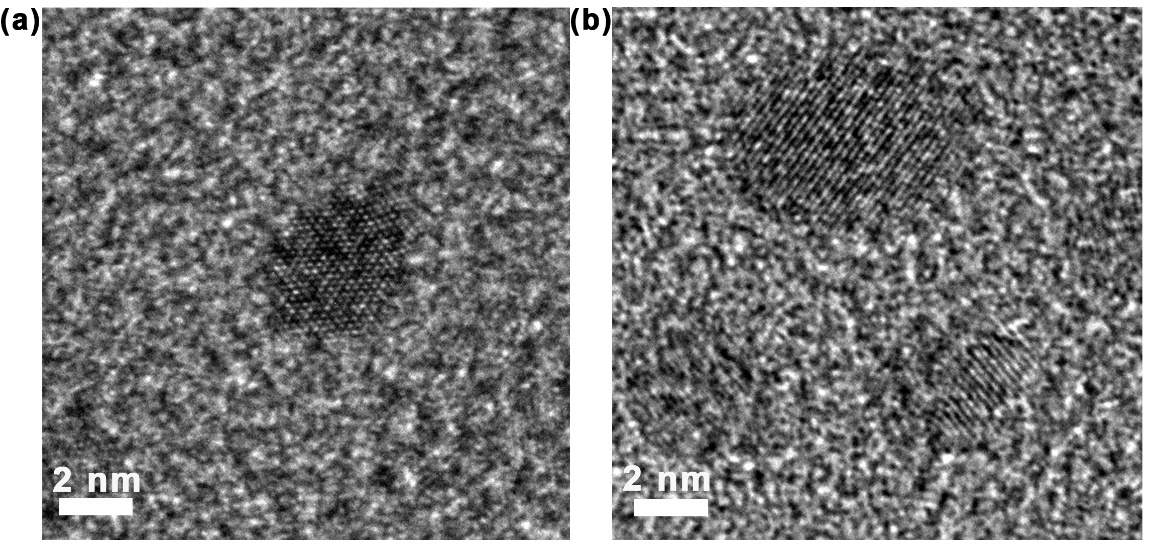


**Figure S4.** HRTEM of S-CDs (a) and N-CDs (b).


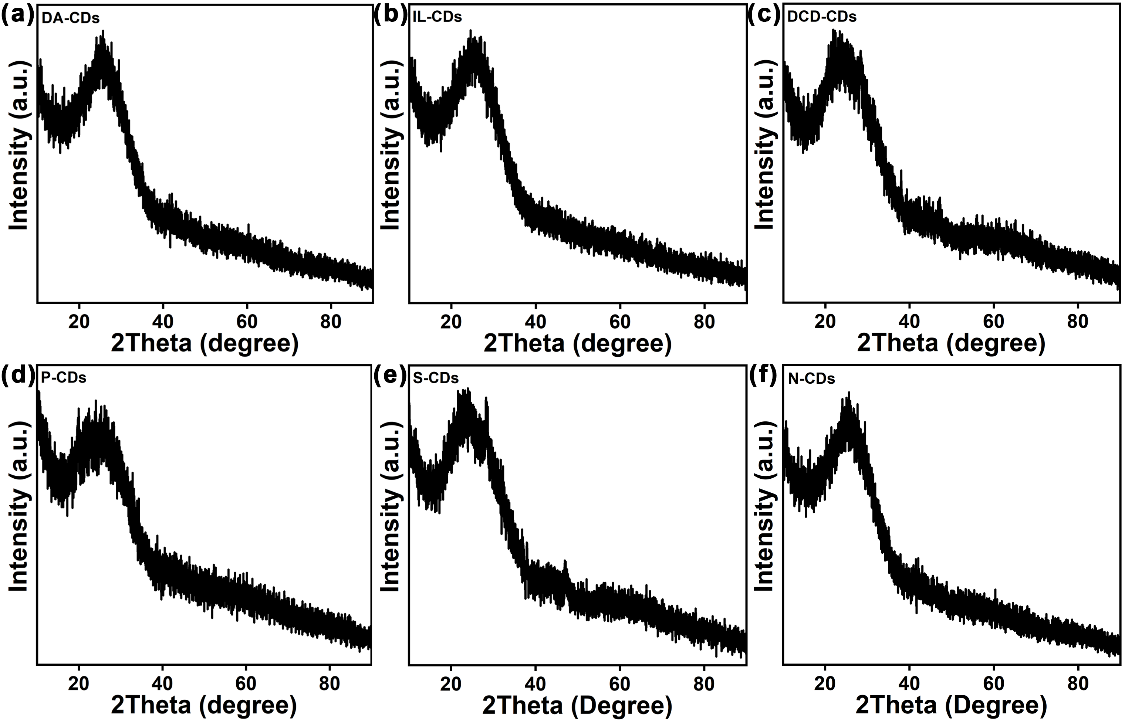


**Figure S5.** XRD patterns of DA-CDs (a), IL-CDs (b), DCD-CDs (c), P-CDs (d), S-CDs (e), and N-CDs (e).


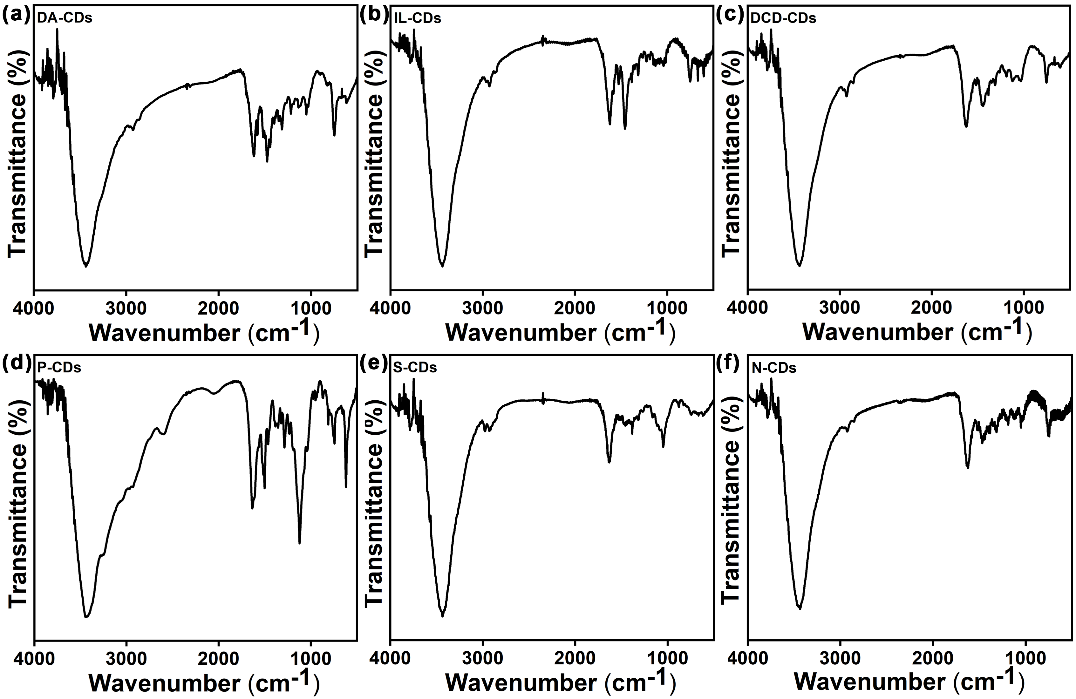


**Figure S6.** FTIR of DA-CDs (a), IL-CDs (b), DCD-CDs (c), P-CDs (d), S-CDs (e), and N-CDs (e).


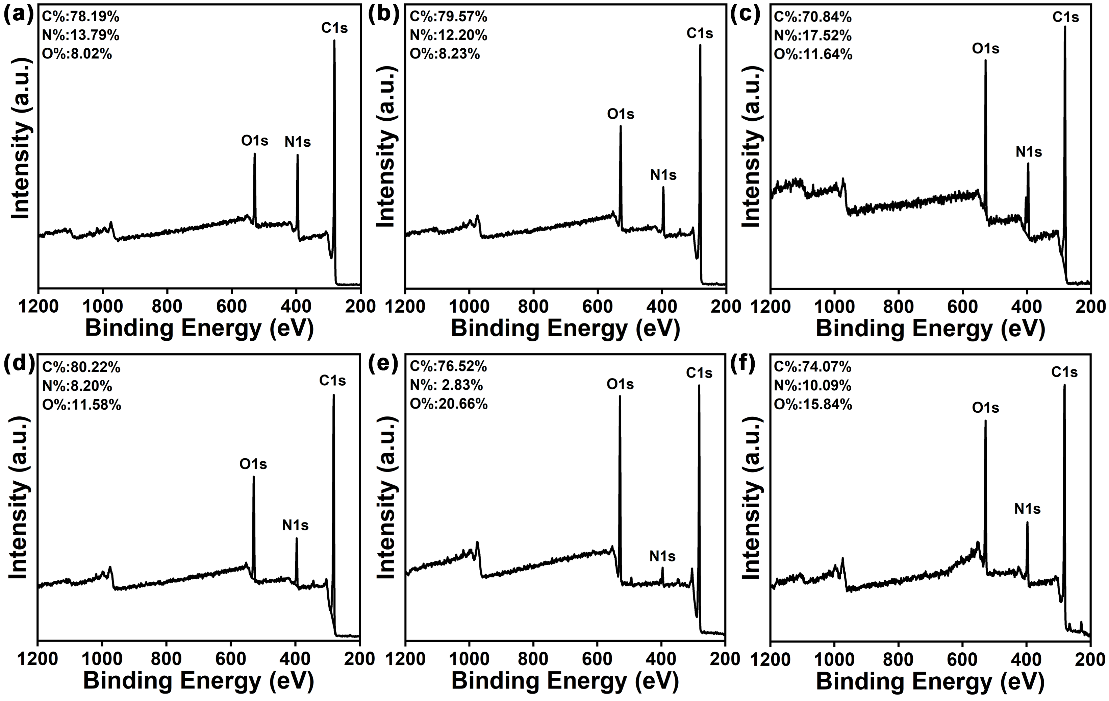


**Figure S7.** XPS spectra and element ratios of DA-CDs (a), IL-CDs (b), DCD-CDs (c), P-CDs (d), S-CDs (e), and N-CDs (e).


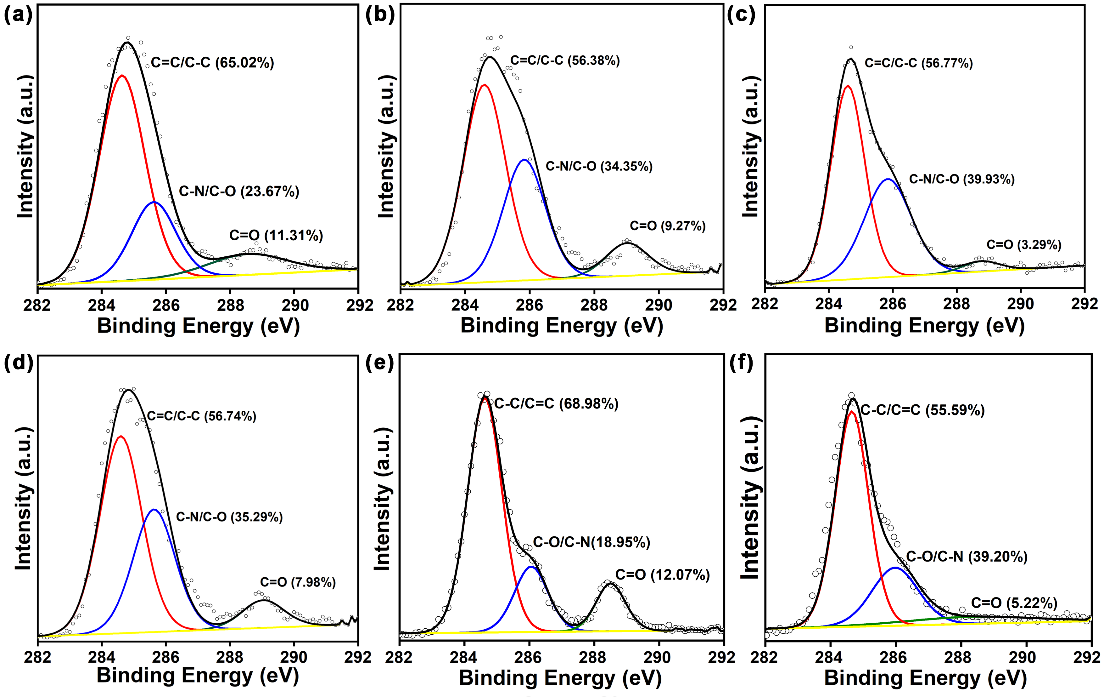


**Figure S8.** C1s spectra and ratio of DA-CDs (a), IL-CDs (b), DCD-CDs (c), P-CDs (d), S-CDs (e), and N-CDs (e).


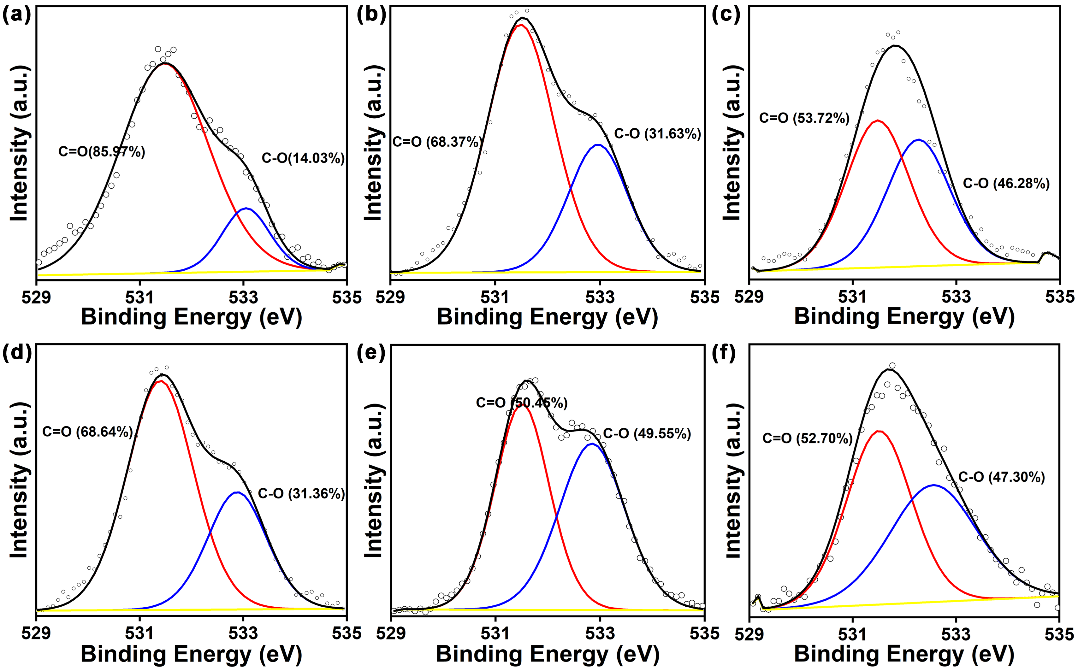


**Figure S9.** O1s spectra and ratio of DA-CDs (a), IL-CDs (b), DCD-CDs (c), P-CDs (d), S-CDs (e), and N-CDs (e).


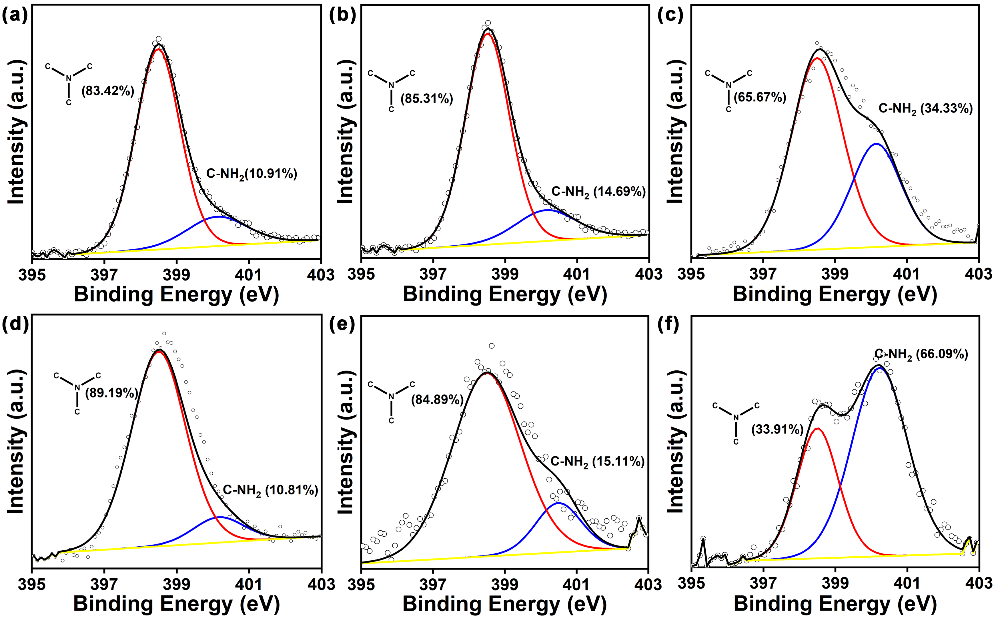


**Figure S10.** N1s spectra and ratio of DA-CDs (a), IL-CDs (b), DCD-CDs (c), P-CDs (d), S-CDs (e), and N-CDs (e).





**Figure S11.** N K-edge absorption spectra of the four selected CDs.


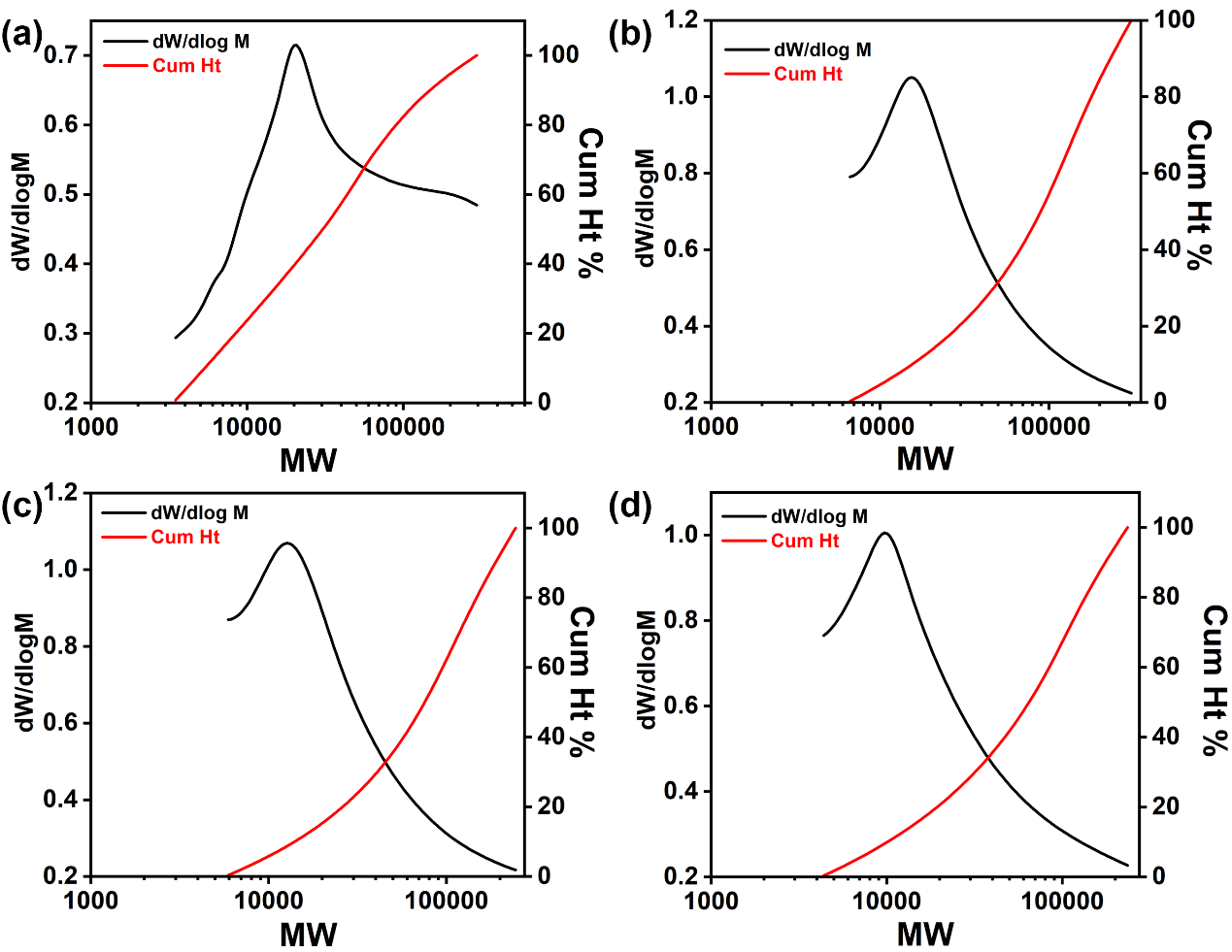


**Figure S12.** Molecular weight distributions of DA-CDs (a), IL-CDs (b), DCD-CDs (c), P-CDs (d).


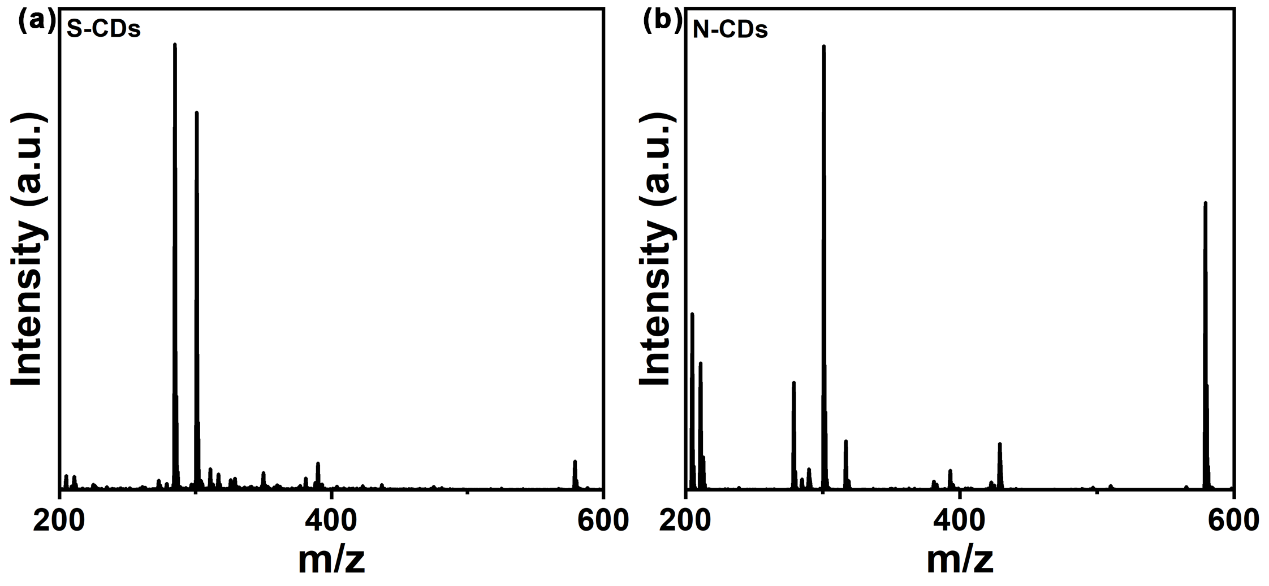


**Figure S13.** Mass spectrometry analysis of S-CDs (a) and N-CDs (b).


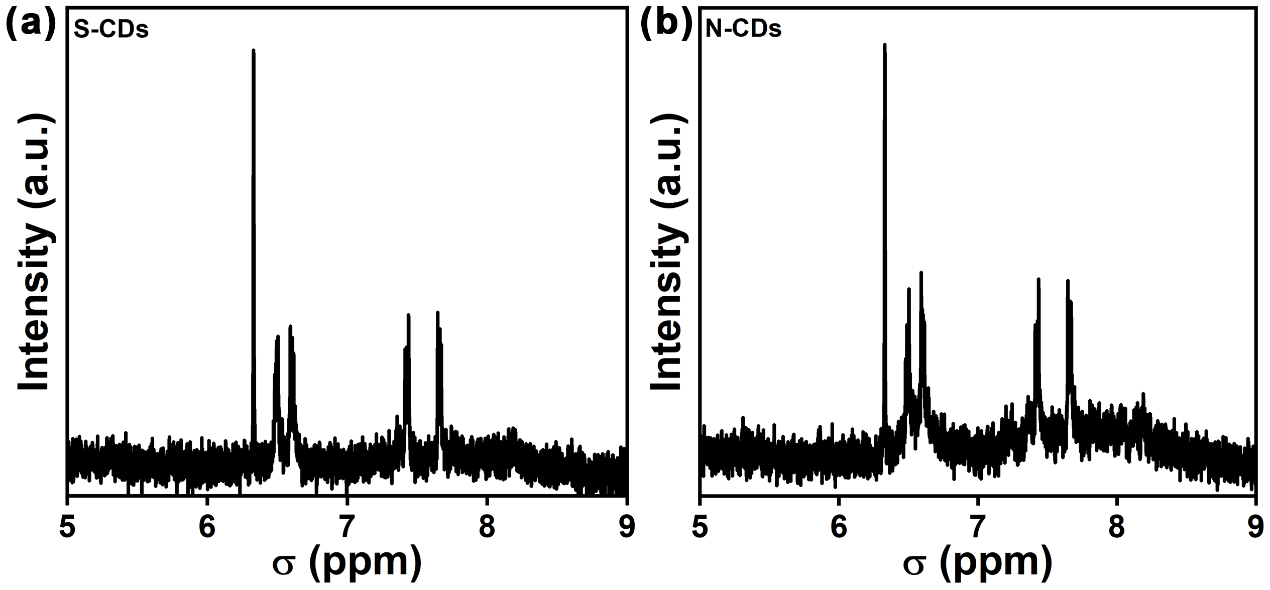


**Figure S14.** ^1^H NMR analysis of S-CDs (b) and N-CDs (b).


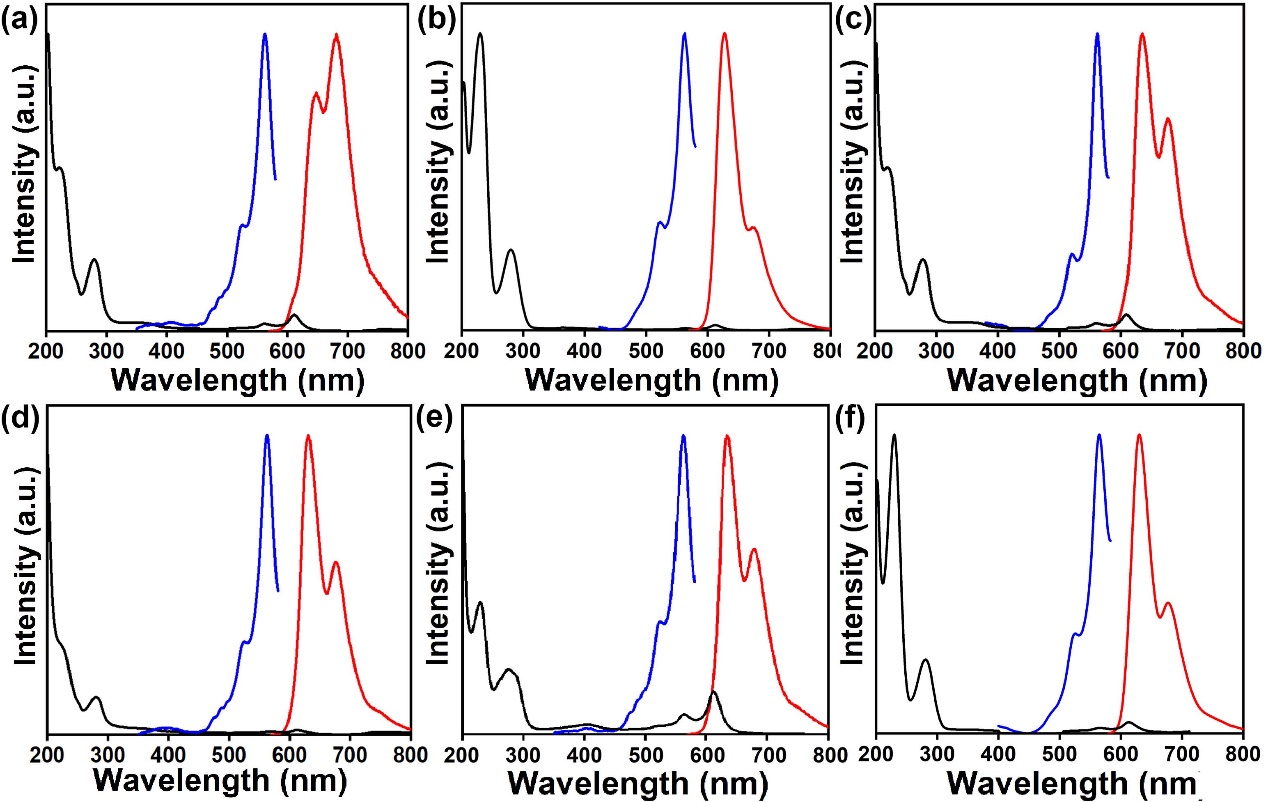


**Figure S15.** Aqueous fluorescence spectra of DA-CDs (a), IL-CDs (b), DCD-CDs (c), P-CDs (d), S-CDs (e) and N-CDs (f). (black line: UV-Vis spectra, blue line: Ex spectra, red line: Em spectra)





**Figure S16.** Fluorescence excitation-dependent spectroscopy in aqueous solution of P-CDs.


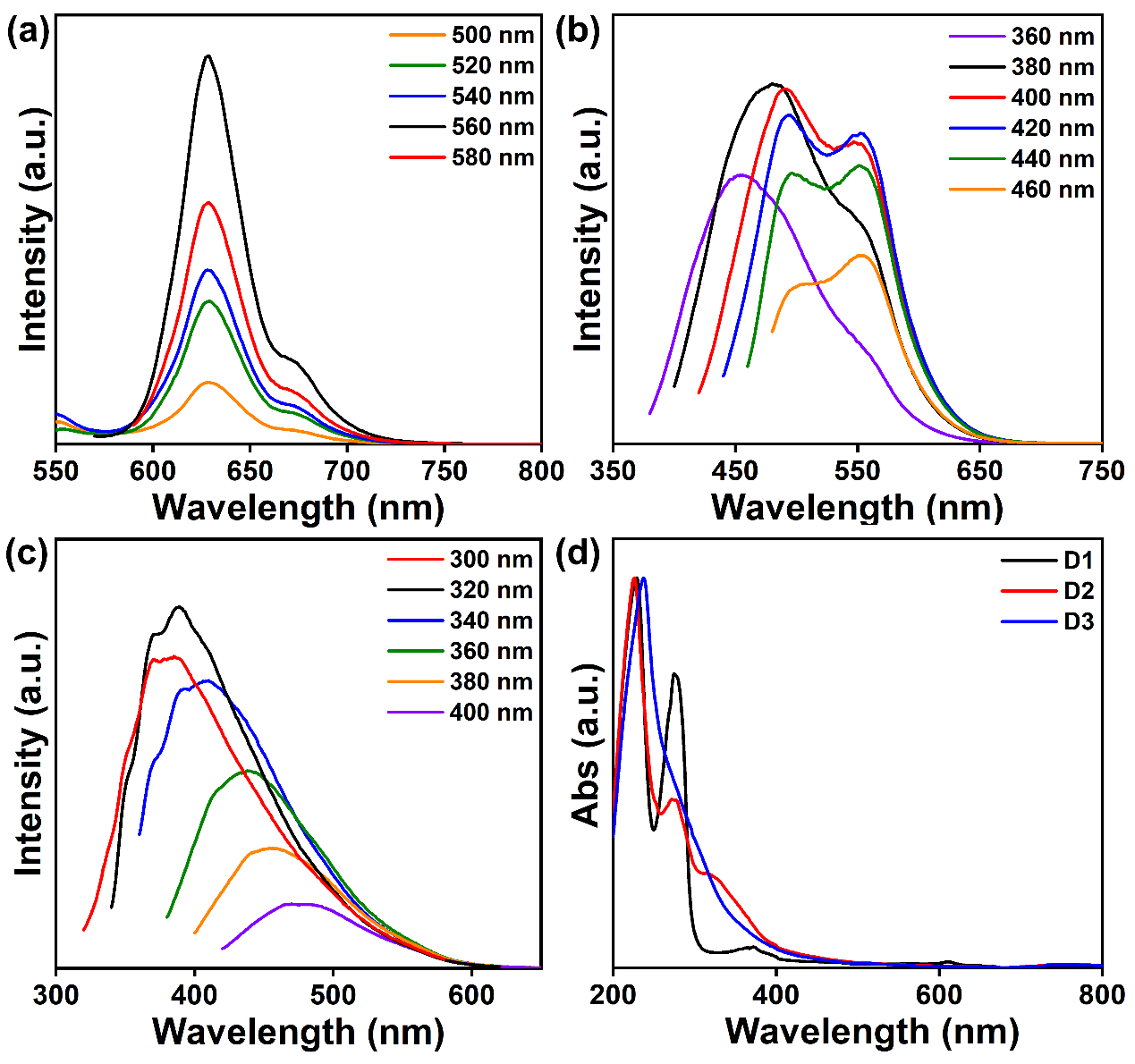


**Figure S17.** PL spectra of D1 (a), D2 (b), and D3 (c). (d) UV–Vis spectra of D1, D2, and D3.


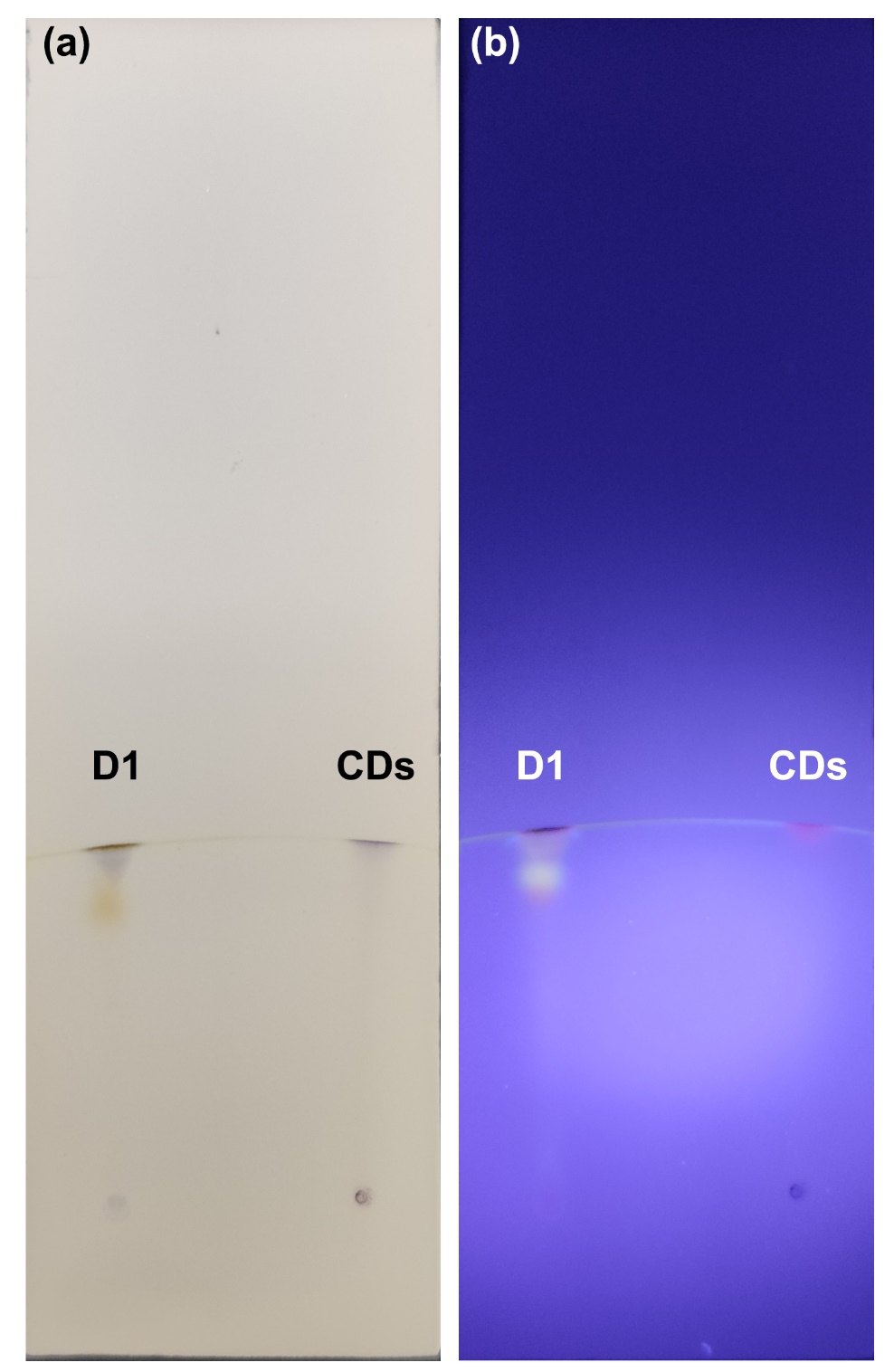


**Figure S18.** Separation of D1 and CDs by TLC in CH_3_CH_2_OH/CH_3_CH_2_OC_2_H_5_.


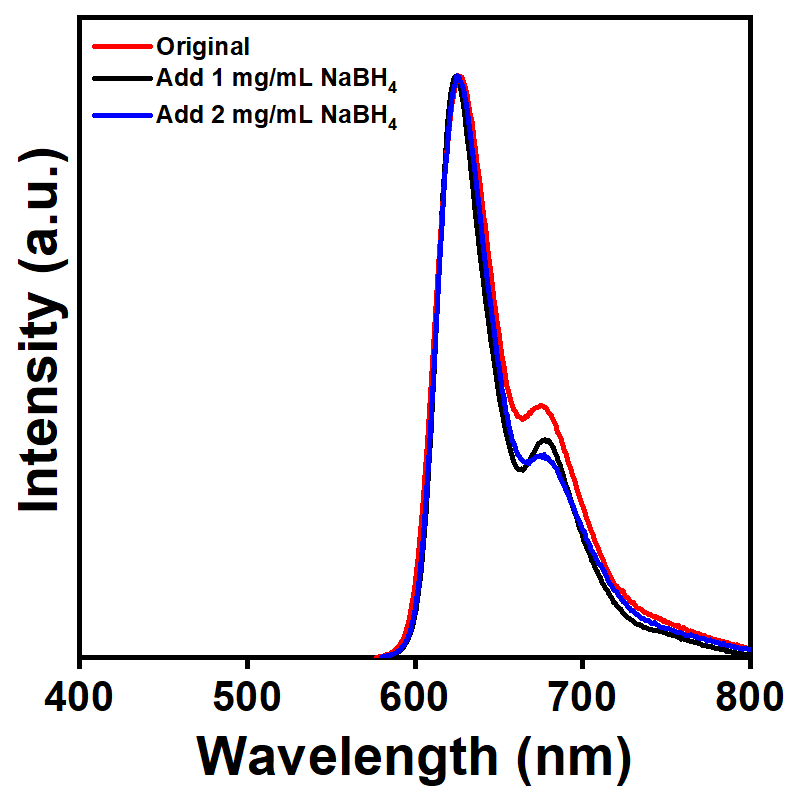


**Figure S19.** PL spectra of the P-CDs in different concentrations of NaBH_4_.





**Figure S20.** PL spectra of the P-CDs in different alkaline environments in EtOH.


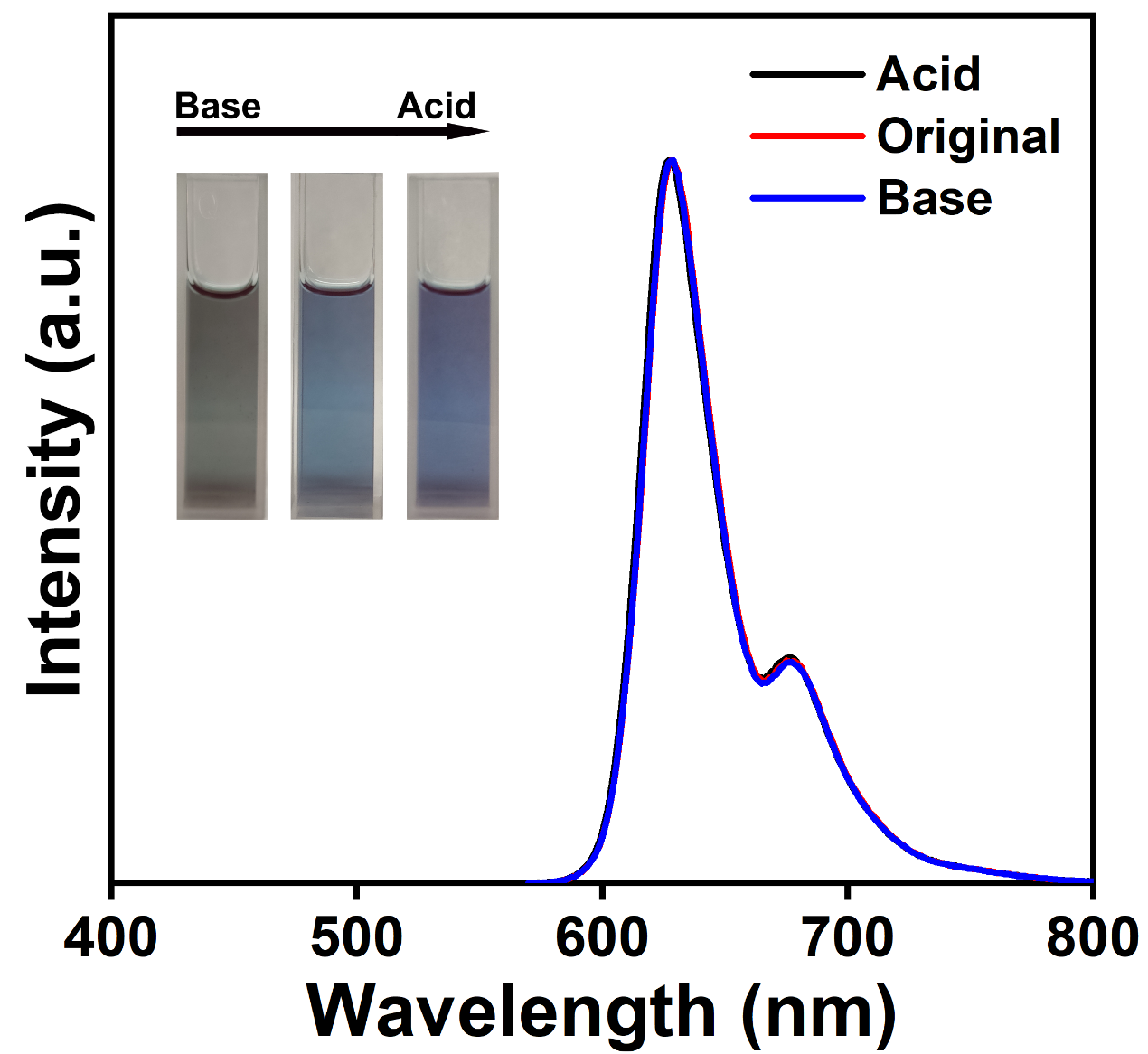


**Figure S21.** PL spectra of the P-CDs in different acid/alkaline environments in aqueous solution.


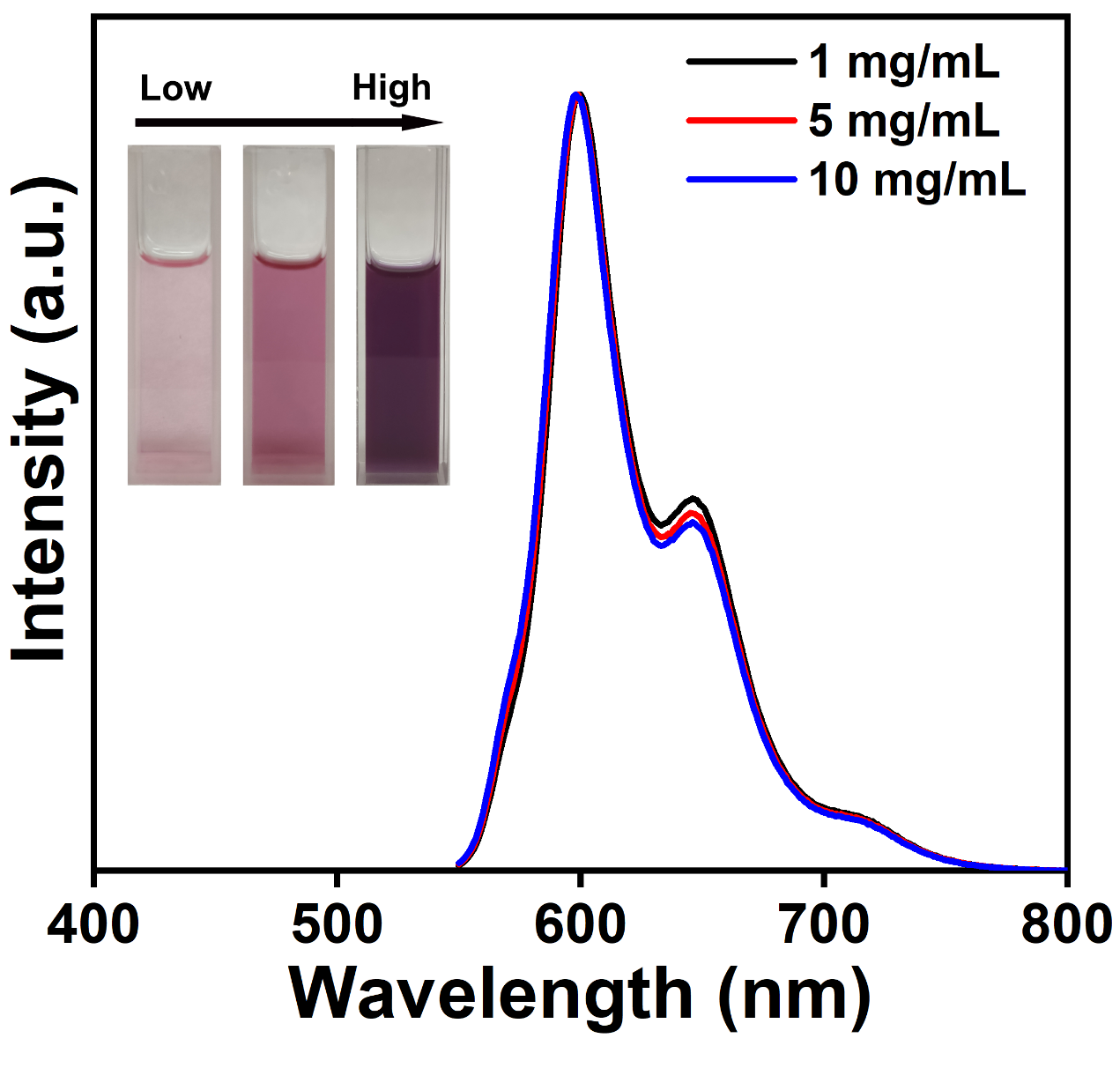


**Figure S22.** PL spectra of the P-CDs in different concentrations in EtOH.





**Figure S23.** Normalization of the third DADS of four selected CDs.


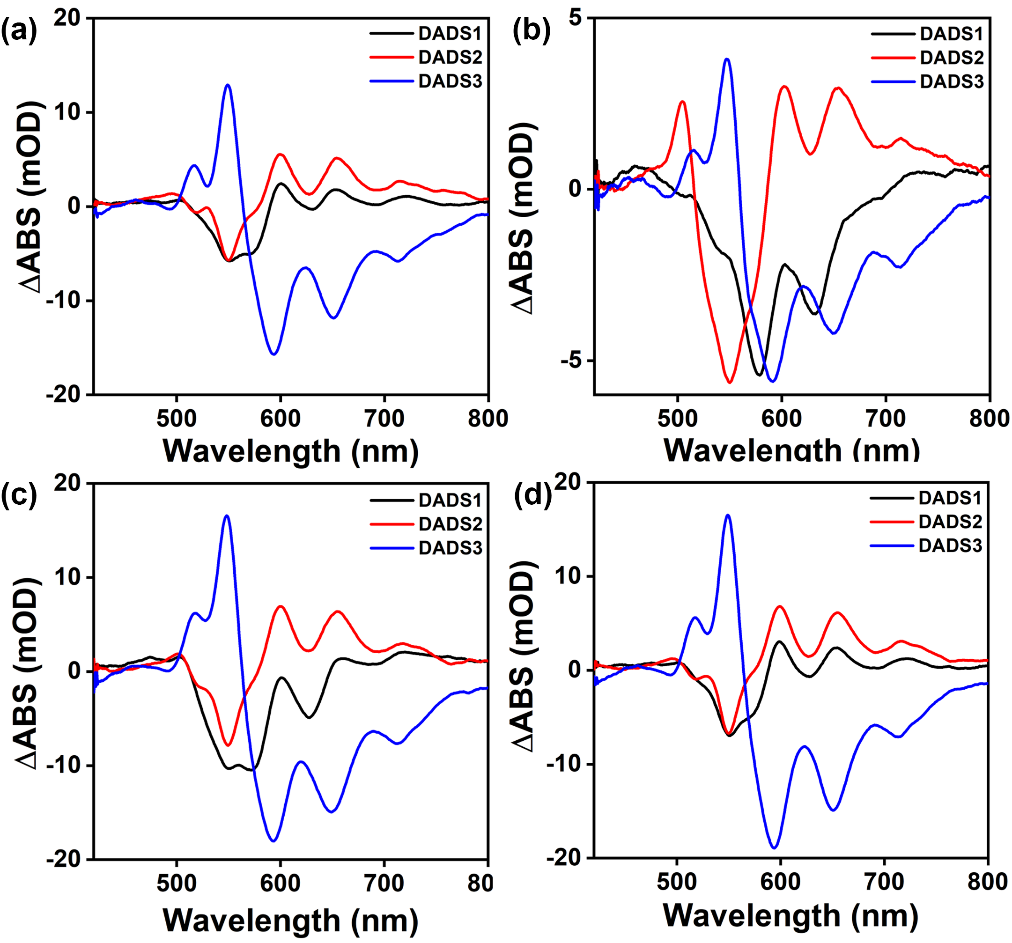


**Figure S24.** Results of the global fitting with three exponential decay functions showing three decay associated difference spectra (DADS), (a) DA-CDs (b) IL-CDs (c) DCD-CDs and (d) P-CDs.





**Figure S25.** Raman spectra of three other CDs and o-phenylenediamine (oPD) excited at 633 nm.


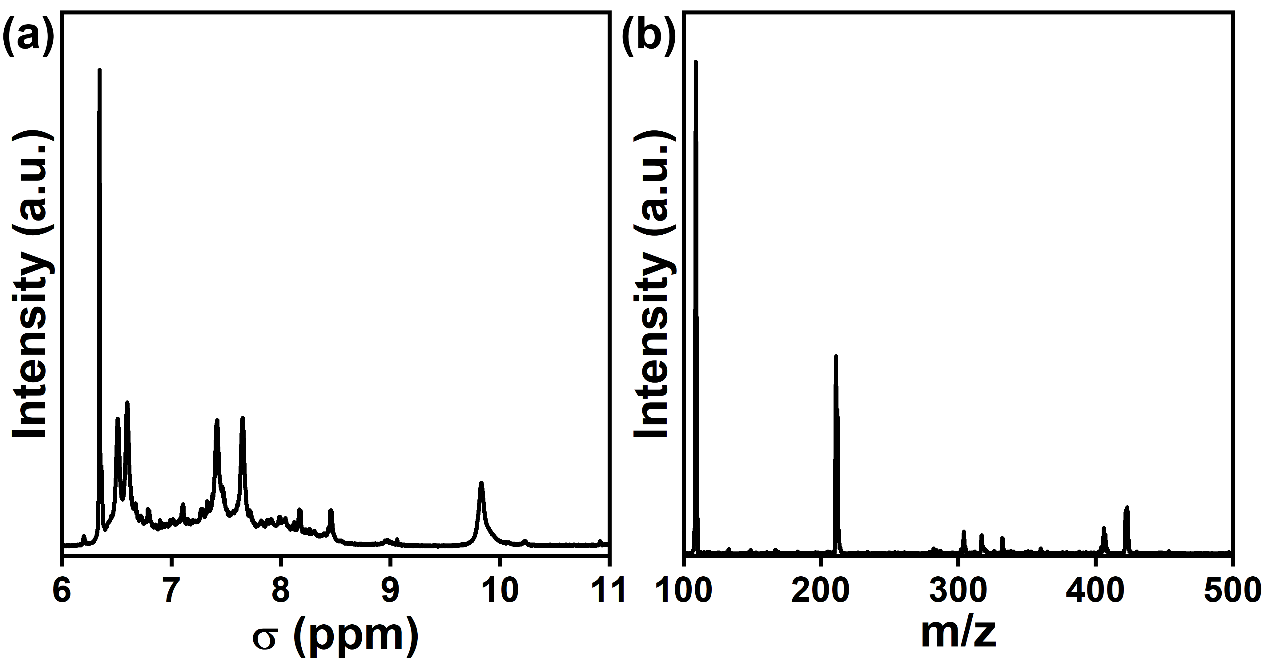


**Figure S26.** ^1^HNMR and MS of PoPD.


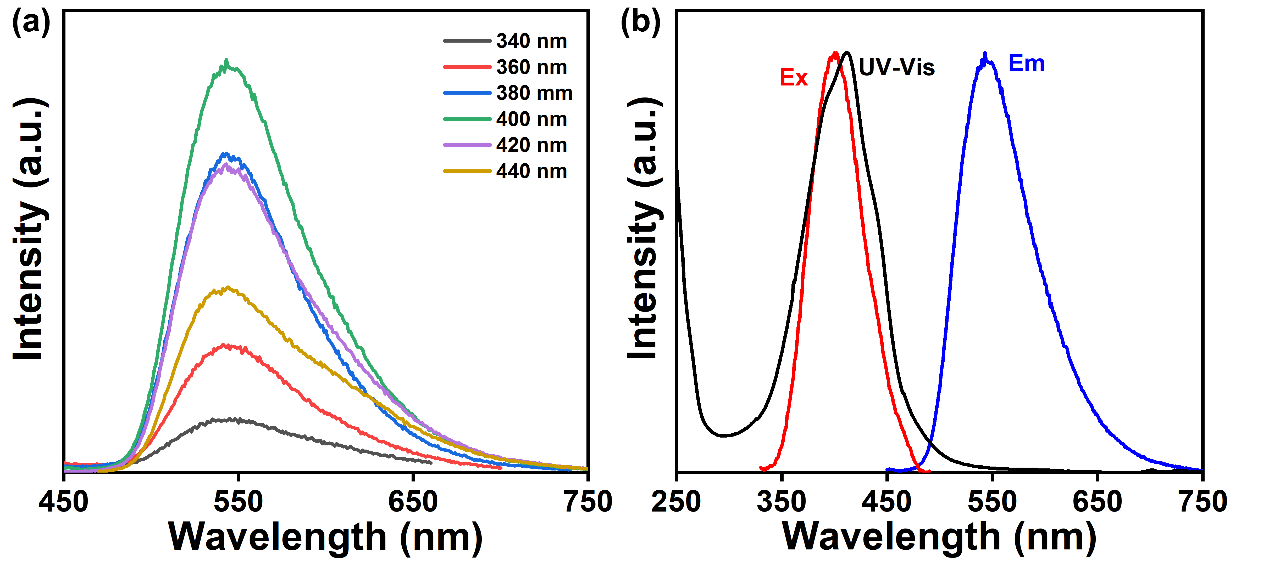


**Figure S27.** (a) Fluorescence excitation-dependent spectroscopy of PoPD; (b) Absorb and fluorescence spectra of PoPD (black line: UV-Vis spectra, red line: Ex spectra, blue line: Em spectra)


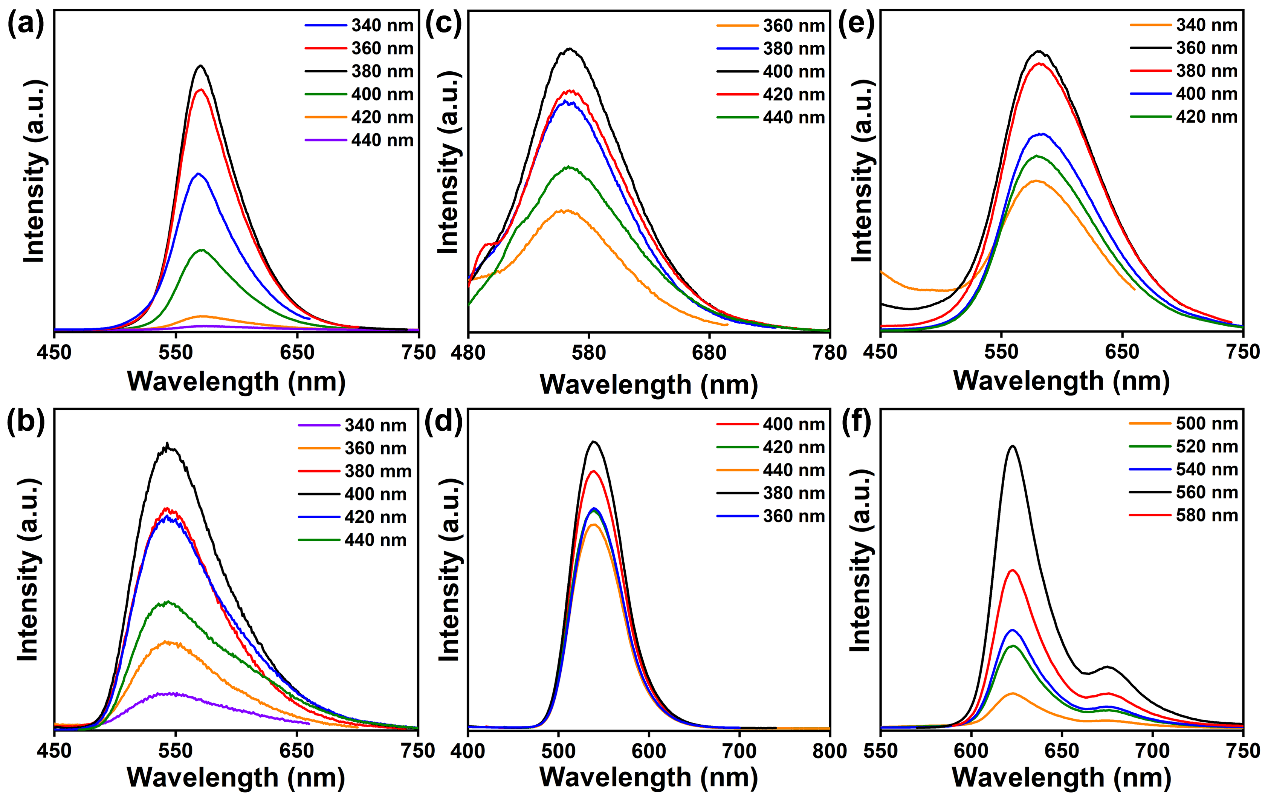


**Figure S28.** PL spectra of PoPD in water (a) and EtoH (b); PL spectra of DPA in water (c) and EtoH (d); PL spectra of DPA-CDs (e) and DPA-CDs-S (f).


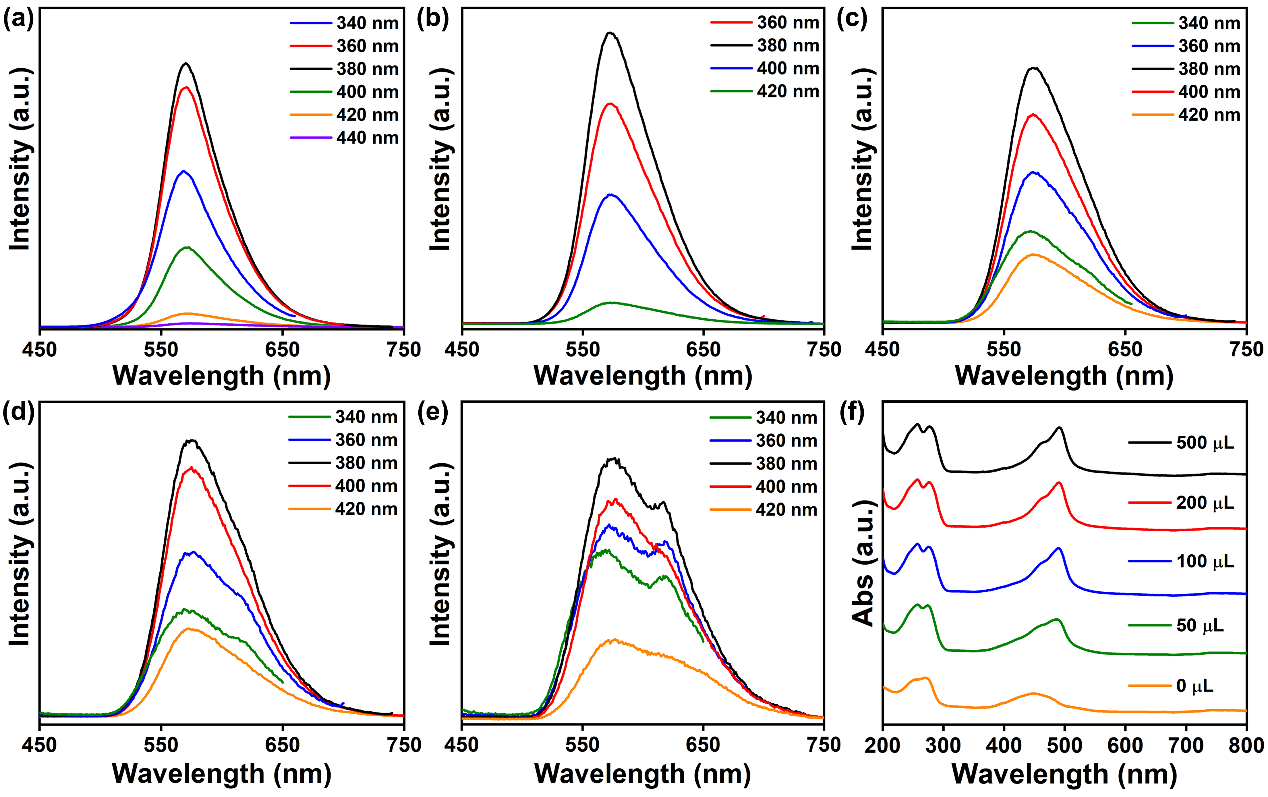


**Figure S29.** PL spectra of PoPD with different concentrations of H_2_SO_4_ in water: (a) 0 μL, (b) 50 μL, (c) 100 μL, (d) 200 μL, and (e) 500 μL. (f) The UV-Vis spectra of PoPD with different concentrations of H_2_SO_4_ in water.


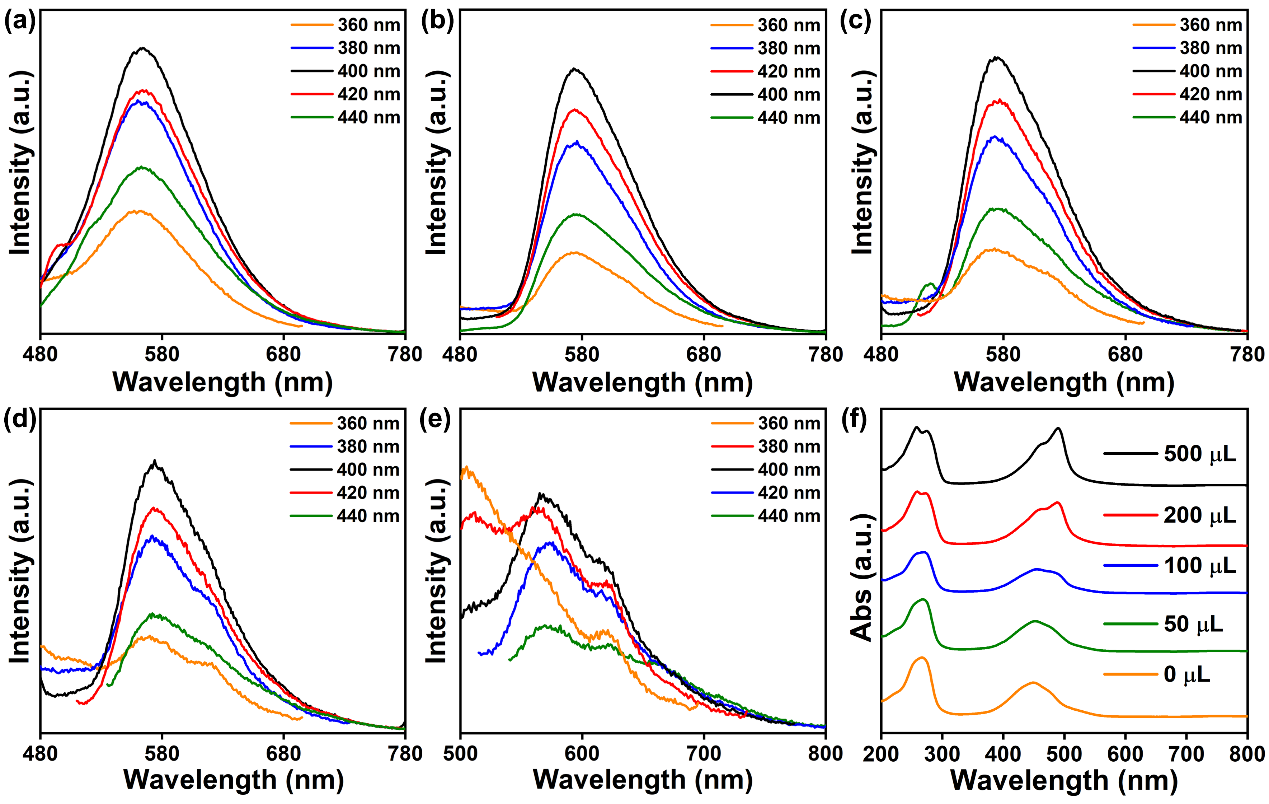


**Figure S30.** PL spectra of DPA with different concentrations of H_2_SO_4_ in water: (a) 0 μL, (b) 50 μL, (c) 100 μL, (d) 200 μL, and (e) 500 μL. (f) UV-Vis spectra of DPA with different concentrations of H_2_SO_4_ in water.


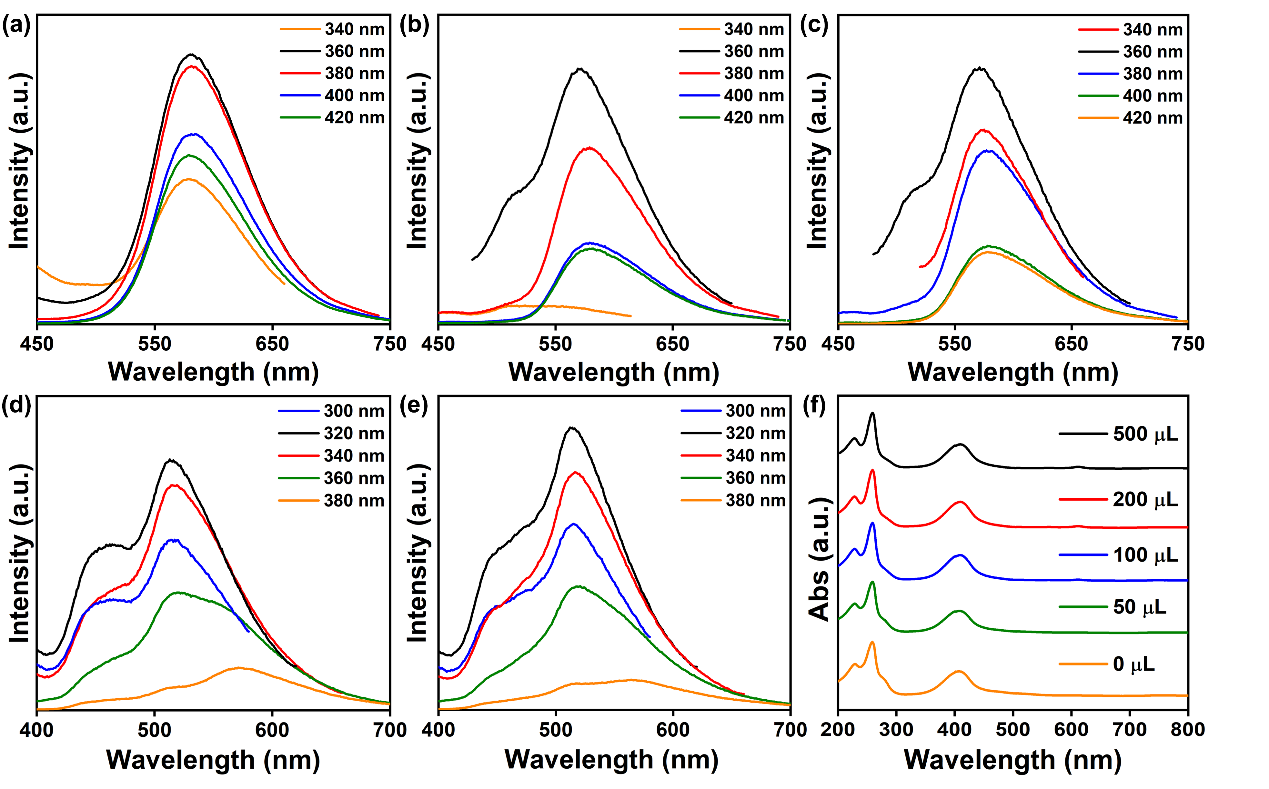


**Figure S31.** PL spectra of DPA-CDs with different concentrations of H_2_SO_4_ in water: (a) 0 μL, (b) 50 μL, (c) 100 μL, (d) 200 μL, and (e) 500 μL. (f) UV-Vis spectra of DPA-CDs with different concentrations of H_2_SO_4_ in water.


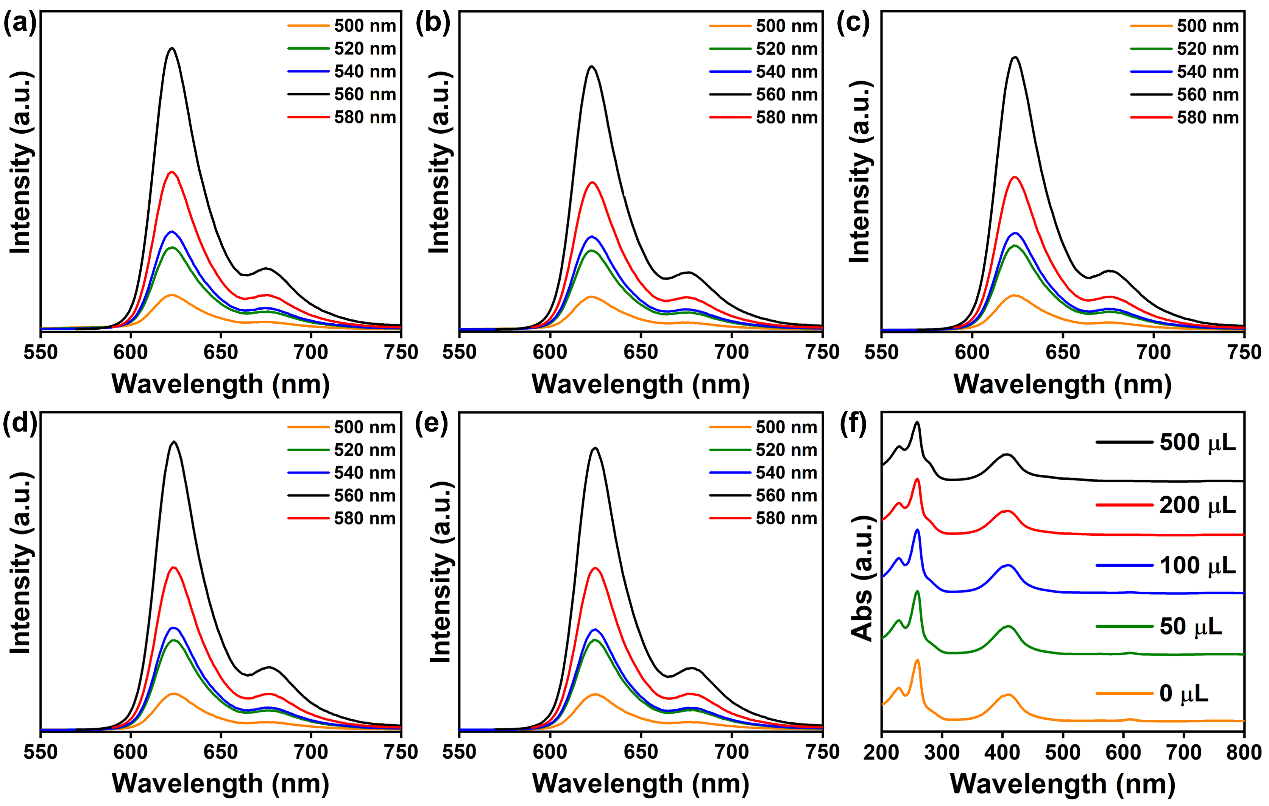


**Figure S32.** PL spectra of DPA-CDs-S with different concentrations of H_2_SO_4_ in water: (a) 0 μL, (b) 50 μL, (c) 100 μL, (d) 200 μL, and (e) 500 μL. (f) UV-Vis spectra of DPA-CDs-S with different concentrations of H_2_SO_4_ in water.


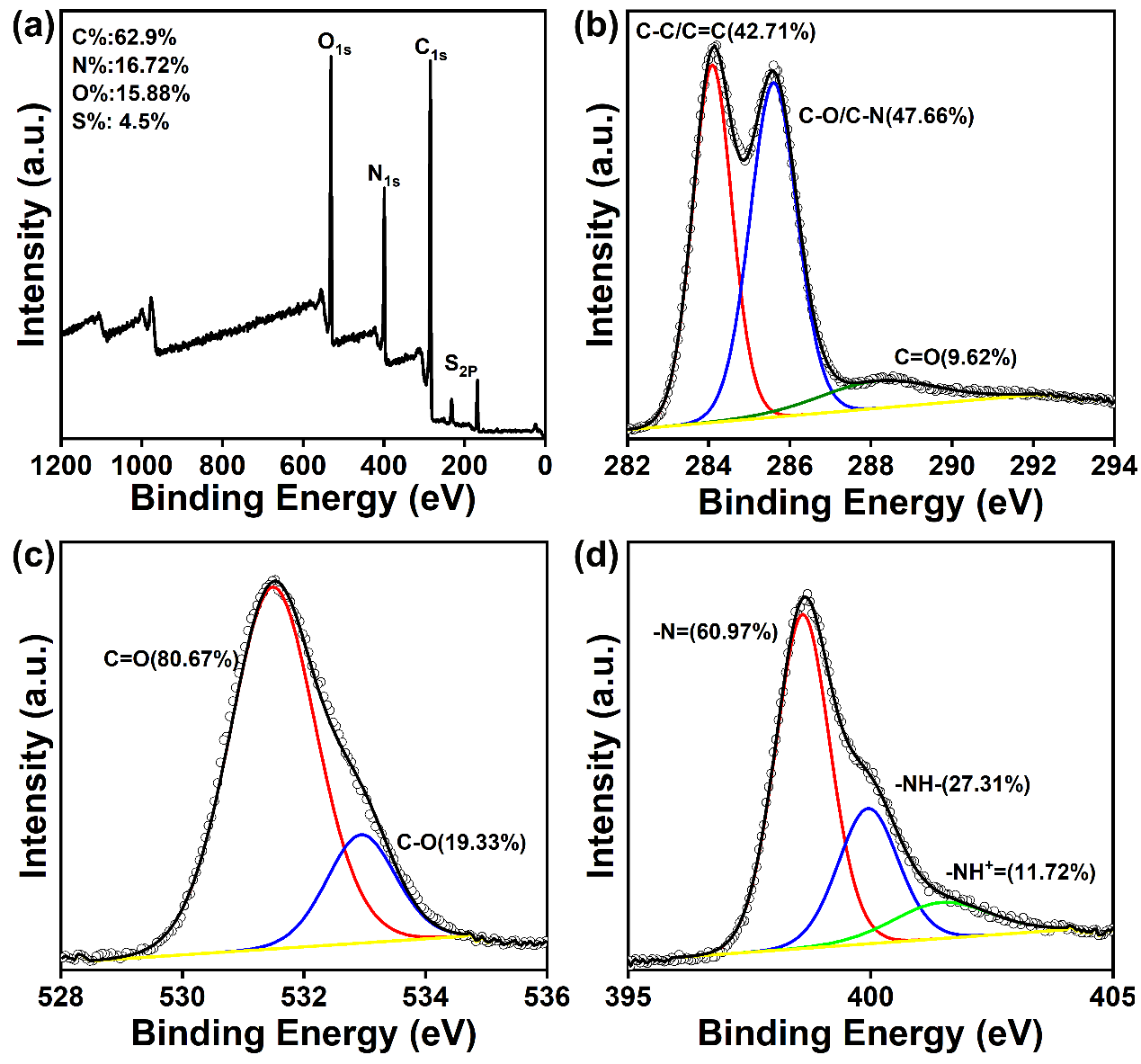


**Figure S33.** XPS spectra (a), C1s (b), O1s (c), and N1s spectrum of PoPD.


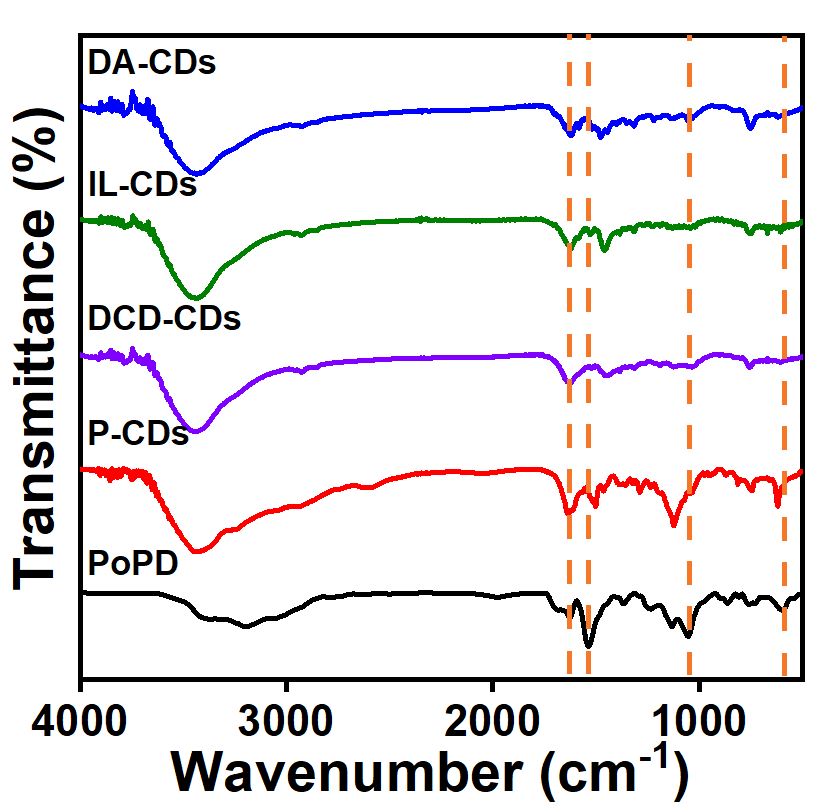


**Figure S34.** FTIR comparison of four selected CDs and PoPD.


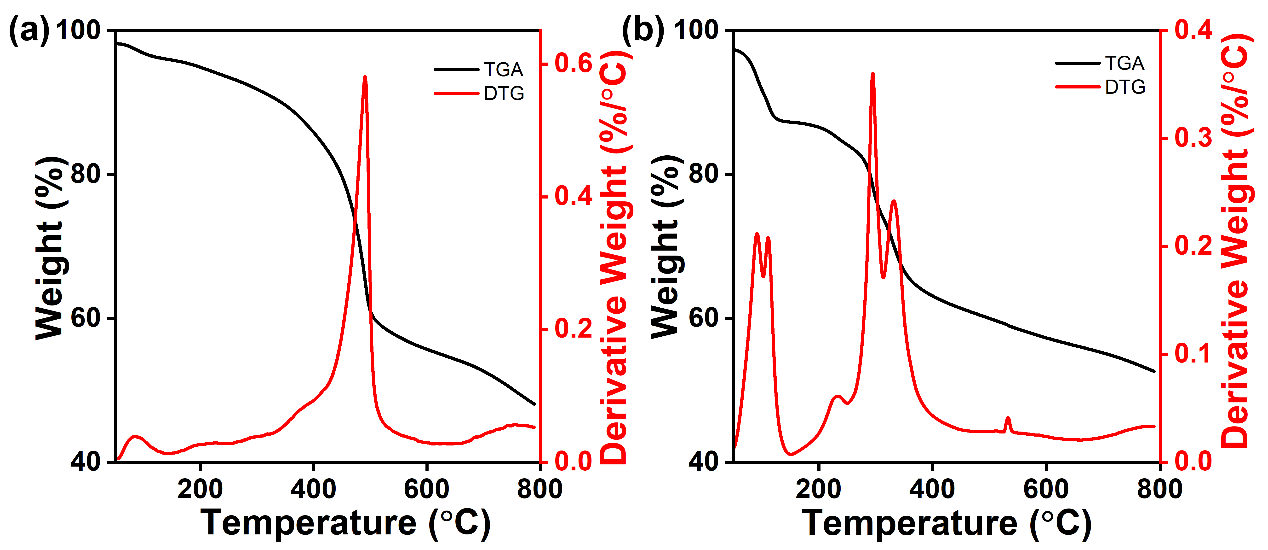


**Figure S35.** DTG and TGA of CDs (a) and PoPD (b).
